# Effectiveness of paediatric occupational therapy for children with disabilities: A systematic review

**DOI:** 10.1111/1440-1630.12573

**Published:** 2019-04-10

**Authors:** Iona Novak, Ingrid Honan

**Affiliations:** ^1^ Cerebral Palsy Alliance Discipline of Child and Adolescent Health The University of Sydney Camperdown North South Wales Australia

**Keywords:** disability, intervention and service provision, occupational therapy, paediatric, systematic review

## Abstract

**Introduction:**

Paediatric occupational therapy seeks to improve children's engagement and participation in life roles. A wide variety of intervention approaches exist. Our aim was to summarise the best‐available intervention evidence for children with disabilities, to assist families and therapists choose effective care.

**Methods:**

We conducted a systematic review (SR) using the Cochrane methodology, and reported findings according to PRISMA. CINAHL, Cochrane Library, MEDLINE, OTSeeker, PEDro, PsycINFO were searched. Two independent reviewers: (i) determined whether studies met inclusion: SR or randomised controlled trial (RCT); an occupational therapy intervention for children with a disability; (ii) categorised interventions based on name, core components and diagnostic population; (iii) rated quality of evidence and determined the strength of recommendation using GRADE criteria; and (iv) made recommendations using the Evidence Alert Traffic Light System.

**Results:**

129 articles met inclusion (*n* = 75 (58%) SRs; *n* = 54 (42%)) RCTs, measuring the effectiveness of 52 interventions, across 22 diagnoses, enabling analysis of 135 intervention indications. Thirty percent of the indications assessed (*n* = 40/135) were graded ‘do it’ (Green Go); 56% (75/135) ‘probably do it’ (Yellow Measure); 10% (*n* = 14/135) ‘probably don't do it’ (Yellow Measure); and 4% (*n* = 6/135) ‘don't do it’ (Red Stop). Green lights were: Behavioural Interventions; Bimanual; Coaching; Cognitive Cog‐Fun & CAPS; CO‐OP; CIMT; CIMT plus Bimanual; Context‐Focused; Ditto; Early Intervention (ABA, Developmental Care); Family Centred Care; Feeding interventions; Goal Directed Training; Handwriting Task‐Specific Practice; Home Programs; Joint Attention; Mental Health Interventions; occupational therapy after toxin; Kinesiotape; Pain Management; Parent Education; PECS; Positioning; Pressure Care; Social Skills Training; Treadmill Training and Weight Loss ‘Mighty Moves’.

**Conclusion:**

Evidence supports 40 intervention indications, with the greatest number at the activities‐level of the International Classification of Function. Yellow light interventions should be accompanied by a sensitive outcome measure to monitor progress and red light interventions could be discontinued because effective alternatives existed.

## Introduction

Occupational therapy intervention for children promotes engagement and participation in children's daily life roles (Mandich & Rodger, 2006). Children's roles include, developing personal independence, becoming productive and participating in play or leisure pursuits (Roger *et al*.). Inability to participate because of disease, disability or skill deficits, can cause marginalisation, social isolation and lowered self‐esteem (Mandich & Rodger, 2006). Occupational therapists select interventions for children based upon an analysis of the child's performance of daily life roles, how their performance is affected by their disability, and how their environment supports or constrains their performance (Mandich & Rodger, 2006).

The practices of paediatric occupational therapists have evolved and changed based on research and theory (Rodger, Brown & Brown, 2005), such as family centred care and the World Health Organisation's (WHO) International Classification of Functioning, Disability and Health (ICF; World Health Organisation, 2001). These frameworks have led many occupational therapists to move away from impairment‐based interventions at the body structures and functions level aimed at remediating the child's deficits (known as ‘bottom‐up’ interventions), and instead to focus on improving functional activity performance and participation (‘top‐down’ interventions) (Weinstock‐Zlotnick & Hinojosa, 2004), as well as partnering with parents to deliver therapy embedded within daily life.

Clinicians will always have different expertise and preferences, but there are financial and ethical ramifications of delivering interventions. Ensuring the latest research findings are easily accessible to families and clinicians is vital. Occupational therapists positively embrace evidence‐based practice, but on the ground, implementation can lag (Flores‐Mateo & Argimon, 2007; Upton, Stephens, Williams & Scurlock‐Evans, 2014). Systematic reviews (SR) indicate that the translation of the latest evidence into routine clinical care lags 10–20 years in all countries and specialities (Flores‐Mateo & Argimon), which for paediatric patients is an entire childhood. Multiple paediatric occupational therapy interventions exist to address children's specific goals. In partnership with parents, it is the therapist's role to choose and tailor the intervention choices to match the child and parent's goals, preferences and potential for improvement based upon their diagnosis. Staying up‐to‐date is time‐consuming. Furthermore, appraising evidence and up skilling in new interventions requires reallocation of time and resources.

The aim of this paper is to systematically describe current intervention options available to paediatric occupational therapists across different child diagnostic populations, rating the quality and recommendations for use of each intervention, using the Grading of Recommendations Assessment, Development and Evaluation (GRADE) system (Guyatt *et al*., 2008) and the Evidence Alert Traffic Light System (Novak & McIntyre, 2010). The purpose of reviewing and rating the entire evidence‐base is to provide a ‘one‐stop’ access guide for clinicians and policy‐makers, allow for the easy comparison of interventions, encourage the uptake of evidence‐based interventions, to confer better outcomes for children. We sought to answer the following ‘PICOs’ question: What is the effectiveness of occupational therapy intervention for children with disabilities? Population = children with a disability (including arthrogyposis OR attention deficit hyperactivity disorder OR autism spectrum disorder OR behaviour disorders OR brachial plexus OR brain injury OR burns OR cerebral palsy OR cancer OR chronic pain OR developmental coordination disorder OR developmental disability OR down syndrome OR fetal alcohol spectrum disorder OR learning disability OR mental health OR muscle diseases; OR intellectual disability OR obesity OR preterm infants OR physical disability OR rheumatoid arthritis OR spina bifida); Intervention = occupational therapy (including all specific named occupational therapy techniques); Comparison = none specified; Outcome = all outcomes accepted; and Study Design = SR OR randomised controlled trials (RCTs).

## Methods

### Study design

A SR of reviews was conducted, to provide an overview of the best available evidence. RCTs not included within the SRs were also appraised.

### Search strategy

This review was carried out according to the Cochrane Collaboration methodology (Higgins & Green, 2011), incorporating the recommended quality features for conducting SRs of reviews (Smith, Devane, Begley & Clarke, 2011), and is reported according to the PRISMA statement (Moher, Liberati, Tetzlaff & Altman, 2010). Relevant articles were identified by searching: CINAHL (1983–2016); Cochrane Database of Systematic Reviews (http://www.cochrane.org); Database of Reviews of Effectiveness (DARE); EMBASE (1980–2016); ERIC; Google Scholar; MEDLINE (1956–2014); OTSeeker (http://www.otseeker.com); and PsycINFO (1935–2016). Searches were supplemented by hand searching and retrieval of any additional articles meeting eligibility criteria that were cited in reference lists. The search of all published studies was performed in March 2014 and updated in August 2018. Interventions and keywords for investigation were identified using the contributing authors’ knowledge.

### Inclusion criteria

Published studies fulfilling the following criteria were included: (i) Type of study: All SRs and RCTs meeting inclusion criteria were appraised. SRs were preferentially sought since they provide a summary of large bodies of evidence and help to explain differences amongst studies. Plus, SRs limit bias. We also included RCTs not included within the SRs, because they are the gold standard design for measuring the effectiveness of interventions. Lower levels of evidence were only included if: the SR reviewed lower levels of evidence; (ii) Types of interventions: Studies that involved the provision of any type of occupational therapy intervention; and (iii) Types of participants: Studies that explicitly involved humans in which 100% of the participants were children of any childhood disability diagnosis.

### Exclusion criteria

(i) Studies about typically developing children or adults; (ii) diagnostic studies OR prognostic studies OR about outcome measure psychometrics OR about theoretical frameworks NOT intervention; (iii) interventions that primarily fall under the skillset of another profession, for example pharmacotherapies, psychotherapy, speech therapies, etc. (iv) a second publication of the same study (Note: RCTs that met inclusion criteria but were also cited within included SRs, were treated as duplicates and not reported on twice); (v) studies were unpublished or non‐peer reviewed; and (vi) full‐text was not available in English.

### Data abstraction

A data abstraction form was devised based on the Cochrane's recommendations (Higgins & Green, 2011). Abstracts identified from searches were screened by two independent raters. Both independent raters reviewed full‐text versions of the articles and articles were retained if they met inclusion criteria. Agreement on inclusion and exclusion assignment was unanimous. Data extracted from included studies comprised: authors and date of study; type of intervention (if named), core components and diagnostic population; who delivered the intervention; location of where the intervention was carried out; intensity of the intervention; study design and original authors’ conclusions about efficacy across study outcomes (Table S1). In addition, based on intervention description and ICF definitions, reviewers assigned an ICF domain to each intervention outcome sought by study authors (World Health Organisation, 2001). Where multiple SRs or RCTs existed, we noted when the older research was superseded by newer evidence. Interventions with the same name and/or similar core components, and that were administered to the same diagnostic populations, were grouped together. All data required to answer the study questions were published within the papers, so no contact with authors was necessary. All the supporting data are included with Table S1.

### Quality of the evidence

Quality ratings were assigned by two independent raters for each publication using GRADE (Guyatt *et al*., 2008), which is endorsed by the World Health Organization. Within GRADE randomised trials are initially rated high, observational studies low; and other levels of evidence very low. However, high quality evidence is downgraded if methodological flaws exist, and low quality evidence is upgraded when high rigor and large effect sizes exist (Guyatt *et al*. ). Ultimately, a high score indicates ‘further research is unlikely to change our confidence in the estimate of effect’; moderate scores indicate ‘further research is likely to have an important impact on our confidence in the estimate of effect and may change the estimate’; low scores indicate ‘further research is very likely to have an important impact on our confidence in the estimate of effect and is likely to change the estimate’; and very low scores indicate ‘any estimate of effect is very uncertain’ (Guyatt *et al*.).

### Strength of recommendation

Unlike SR frameworks, the GRADE framework does not solely examine effect size to determine efficacy of intervention. Instead, effect size makes up just one component when weighing up the benefits and harms of each intervention. In line with the GRADE framework, the following factors were considered by both independent raters when evaluating the body of evidence for the intervention and arriving at a strength of recommendation for each diagnostic group: (i) methodological quality regarding likely benefits vs. likely risks; (ii) inconvenience; (iii) importance of the outcome that the intervention prevents; (iv) magnitude of intervention effect (effect size); (v) precision of estimate of effect; (vi) burdens; (vii) costs; and (viii) varying clinician and family values (Guyatt *et al*., 2008).

The *Evidence Alert Traffic Light System* (Novak & McIntyre, 2010) was then applied based on the strength of recommendations by both independent raters. The Evidence Alert Traffic Light System is a GRADE‐complementary knowledge translation tool designed to assist clinicians and families to obtain easily readable, clinically useful answers within minutes (Campbell, Novak, McIntyre & Lord, 2013), because the alert uses a simple, three‐level colour coding that recommends a course of action. Green signifies ‘go’ because high quality evidence indicates effectiveness; red signifies ‘stop’ because high quality evidence indicates harm or ineffectiveness; and yellow signifies ‘measure’ because insufficient evidence exists to be certain about whether the child will benefit. Yellow can be assigned in three scenarios: (i) promising evidence (weak positive), (ii) unknown effectiveness because no research exists, or (iii) evidence suggests possibly no effect (weak negative).

### Ethics and data

The study did not involve contact with humans, so the need for ethical approval was waived by the Cerebral Palsy Alliance's National Health and Medical Council Human Research Ethics Committee. This SR was not registered.

## Results

**Figure 1:**
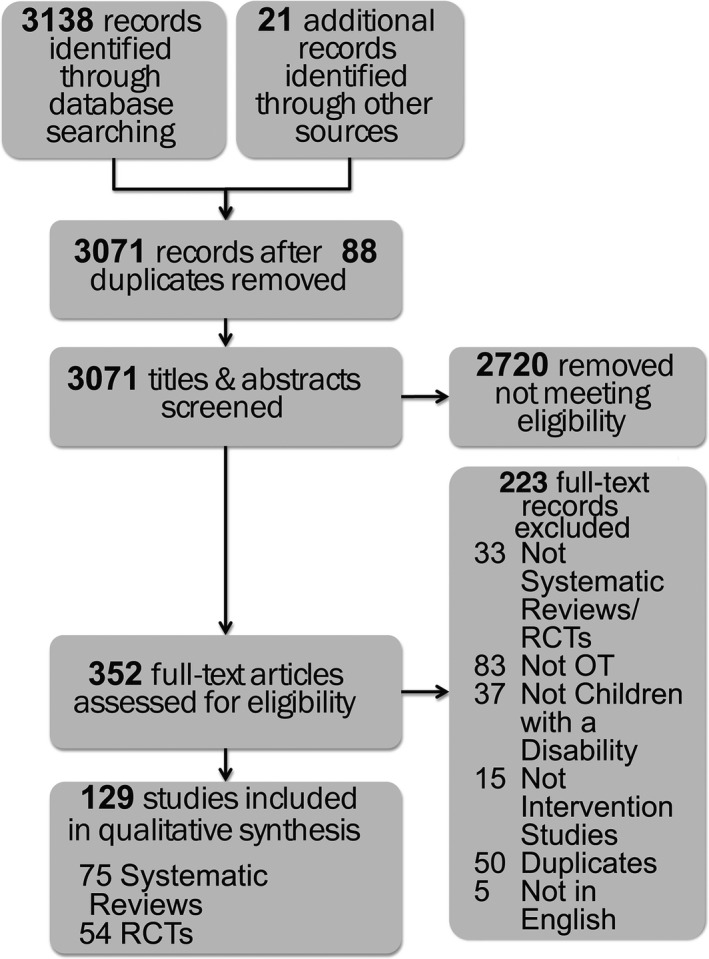
PRISMA Flow Diagram

3138 citations were identified using the search strategy, of which 129 articles met the inclusion criteria for review. Of the 129 included articles, 58% (*n* = 75/129) were SRs; 42% (*n* = 54/129) were RCTs. Note, more than 54 RCTs exist in the paediatric occupational therapy evidence base, but we treated any RCT that was cited within an included SR as a duplicate. Flow of information is presented in the PRISMA diagram (Fig. 1).

The results are now presented using PICO question format headings.

### **P**opulation (Participants)

Included studies were across the following childhood disability diagnoses: arthrogyposis; attention deficit hyperactivity disorder (ADHD); autism spectrum disorder (ASD); behaviour disorders; brachial plexus injury; brain injury (BI); burns; cerebral palsy (CP); cancer; chronic pain; developmental coordination disorder (DCD); developmental disability (DD); Down syndrome; foetal alcohol spectrum disorder; learning disability (LD); mental health; intellectual disability (ID); obesity; preterm infants; physical disability; rheumatoid arthritis; and spina bifida. Some studies included samples from a variety of the aforementioned diagnoses. Consistent with childhood disability population incidence data, more research existed about ASD (*n* = 32/135; 24%), ADHD (*n* = 8/135; 6%), CP (*n* = 38/135; 28%) and DCD (*n* = 9/135; 7%), than other conditions.

Paediatric occupational therapy involves working with the child, the parent and the family unit: The child was the primary client for 87% (*n* = 45/52) of the interventions, i.e. therapy focussed on improving the child outcomes (e.g. an orthotic worn by the child to improve hand function), whereas the parent was the primary client for 13% (*n* = 7/52) for the interventions (e.g. parent education, aiming to improve knowledge, skills and confidence).

### **I**nterventions

Included studies, researched the effectiveness of 52 occupational therapy intervention groups: (1) Acupuncture; (2) Assistive Devices; (3) Assistive Technology; (4) Behavioural Interventions including Applied Behavioural Analysis (ABA) and Positive Parenting Program (Triple P); (5) Bimanual Training; (6) Biofeedback; (7) Coaching; (8) Cognitive Interventions including CogFun, CogMed, (9) Cognitive Orientation to Occupational Performance (CO‐OP); (10) Conductive Education; (11) Constraint Induced Movement Therapy (CIMT); (12) CIMT &/or Bimanual; (13) Context Focused; (14) Ditto™ (hand held education & distraction device for burns patients); (15) Early Intervention, including a Developmental Approach, Neurodevelopmental Therapy (NDT) and Goals Activity and Motor Enrichment (GAME); (16) Electrical Stimulation (ES); (17) Family Centred Care; (18) Feeding Interventions; (19) Goal Directed Training, including Task Specific Training, Functional Training, Neuromotor Task Training (NTT) and Motor Imagery; (20) Handwriting Interventions; (21) Hippotherapy [Therapeutic Horse Riding]; (22) Home Programs; (23) Joint Attention; (24) Massage; (25) Meditation and/or Mindfulness; (26) Mental Health Interventions; (27) Neuro‐Developmental Therapy (NDT); (28) Occupational Therapy after BoNT; (29) Orthotics; (30) Pain Management; (31) Parent Counselling; (32) Parent Education/Parent Training; (33) Picture Exchange Communication System (PECS); (34) Play Therapy; (35) Positioning; (36) Pressure Care; (37) School Therapy; (38) Self‐Management; (39) Sensation Training; (40) Sensory Approach, including brushing, therapy balls, weighted vests, warm‐ups, sensory stimulation; (41) Sensory Integration, including sensory diets, swinging, brushing, therapy balls, weighted vests, body socks; (42) Skills Training via Mental Imagery; (43) Sleep Interventions; (44) Social Skills Training; (45) Stretching, including passive: self‐administered, therapist‐administered and device‐administered; (46) Treatment and Education of Autistic and Communication Handicapped Children (TEACCH); (47) Therapeutic Listening; (48) Treadmill Training; (49) Visual Motor Interventions; (50) Weight Loss; (51) Whole Body Vibration; and (52) Yoga.

### **O**utcomes

Of the 12 included articles, authors measured the effectiveness of 52 occupational therapy interventions, across 22 diagnoses. From this, 136 intervention outcome indicators were identified, whereby an intervention, with an individual target outcome was administered to specific diagnostic groups. Insufficient data was available for analysis on one of these outcome indicators (number 74 in Table S1, where the SR authors found no publish data examining the effectiveness of hand orthotics in children with brain injury and therefore no recommendations could be made), (Jackman, Novak & Lannin, 2014) resulting in 135/136 intervention outcome indicators available for analysis.

Of the 135 intervention outcome indications: 30% (*n* = 40/135) were graded ‘do it’ (Green Go) (Arbesman, Bazyk & Nochajski, 2013; Bellows *et al*., 2011; Bleyenheuft, Arnould, Brandao, Bleyenheuft & Gordon, 2015; Brown, Kimble, Rodger, Ware & Cuttle, 2014; Chang & Yu, 2014; Chen, Pope, Tyler & Warren, 2014c; Chen *et al*., 2014b; Christmas, Sackley, Feltham & Cummins, 2018; Crompton *et al*., 2007; Estes *et al*., 2014; Fehlings *et al*., 2010; Frolek Clark & Schlabach, 2013; Hechler *et al*., 2014; Heinrichs, Kliem & Hahlweg, 2014; Hoare & Imms, 2004; Hoare, Imms, Carey & Wasiak, 2007; Hoare *et al*., 2010; Hoy, Egan & Feder, 2011; Huang, Fetters, Hale & McBride, 2009; Inguaggiato, Sgandurra, Perazza, Guzzetta & Cioni, 2013; Kamps *et al*., 2015; Kasari *et al*., 2016; Kaya Kara *et al*., 2015; Kurowski *et al*., 2014; Lannin, Scheinberg & Clark, 2006; Lidman, Nachemson, Peny‐Dahlstrand & Himmelmann, 2015; Lin & Wuang, 2012; Madlinger‐Lewis *et al*., 2014; Maeir *et al*., 2014; Novak, 2014a; Park, Maitra, Achon, Loyola & Rincón, 2014; Speth *et al*., 2015; Spittle, Orton, Anderson, Boyd & Doyle, 2012; Spittle, Orton, Doyle & Boyd, 2007; Stavness, 2006; Stickles Goods, Ishijima, Chang & Kasari, 2013; Vroland‐Nordstrand, Eliasson, Jacobsson, Johansson & Krumlinde‐Sundholm, 2016; Zwaigenbaum *et al*., 2015); 56% (75/135) were graded ‘probably do it’ (Yellow Measure) (Armstrong, 2012; Au *et al*., 2014; Auld, Russo, Moseley & Johnston, 2014; Bialocerkowski, Kurlowicz, Vladusic & Grimmer, 2005; Bodison & Parham, 2018; Cameron *et al*., 2017a, 2017b; Chacko *et al*., 2014; Chantry & Dunford, 2010; Chen, Lee & Howard, 2014a; Chiu, Ada & Lee, 2014; Cole, Harris, Eland & Mills, 1989; Copeland *et al*., 2014; Dagenais *et al*., 2009; De Vries, Beck, Stacey, Winslow & Meines, 2015; Duncan *et al*., 2012; Fedewa, Davis & Ahn, 2015; Grynszpan, Weiss, Perez‐Diaz & Gal, 2014; Hahn‐Markowitz, Berger, Manor & Maeir, 2017; Hammond, Jones, Hill, Green & Male, 2014; Huang *et al*., 2014; Jackman *et al*., 2018; James, Ziviani, Ware & Boyd, 2015; Janeslätt, Kottorp & Granlund, 2014; Jones *et al*., 2014; Krisanaprakornkit, Ngamjarus, Witoonchart & Piyavhatkul, 2010; Lannin, Novak & Cusick, 2007; Malow *et al*., 2014; Maskell, Newcombe, Martin & Kimble, 2014; Mast *et al*., 2014; Matute‐Llorente, González‐Agüero, Gómez‐Cabello, Vicente‐Rodríguez & Mallén, 2014; McLean *et al*., 2017; Meany‐Walen, Bratton & Kottman, 2014; Miller‐Kuhaneck & Watling, 2018; Montero & Gómez‐Conesa, 2014; Morgan, Novak, Dale & Badawi, 2015; Morgan *et al*., 2016a; Morgan, Novak, Dale, Guzzetta & Badawi, 2016b; Pfeiffer B & Arbesman, 2018; Polatajko & Cantin, 2010; Reeuwijk, van Schie, Becher & Kwakkel, 2006; Schaaf, Dumont, Arbesman & May‐Benson, 2018; Smith *et al*., 2014; Snider, Majnemer & Darsaklis, 2010; Storebø *et al*., 2011; Tatla *et al*., 2013; Tatla, Sauve, Jarus, Virji‐Babul & Holsti, 2014; Vargas & Lucker, 2016; Westendorp *et al*., 2014; Whalen & Case‐Smith, 2012; Xu, He, Mai, Yan & Chen, 2015; Zadnikar & Kastrin, 2011; Ziviani, Feeney, Rodger & Watter, 2010; Zwicker & Mayson, 2010); 10% (*n* = 14/130) were graded ‘probably don't do it’ (Yellow Measure) (Wallen & Gillies, 2006; Wells, Marquez & Wakely, 2018); and 4% (*n* = 6/135) were graded ‘don't do it’ (Red Stop) (Gringras *et al*., 2014; Katalinic *et al*., 2010).

The 40 green light ‘do it’ interventions indications included: (1) Behavioural Intervention using ABA for children with ASD; (2) Behavioural Intervention using Triple P for children behaviour disorders; (3) Behavioural Intervention using token economy contracts for children with a brain injury; (4) Bimanual Training for children with hemiplegic CP; (5) Coaching for parents of children at risk of disability to promote development; (6) Coaching for parents of children with ASD to promote function and behaviour; (7) CAPS cognitive intervention for children with brain injury to improve long term executive function; (8) Cog‐Fun intervention for children with attention deficit disorder to improve executive function; (9) CO‐OP for children with DCD for functional motor task performance; (10) CIMT for children with CP to improve hand function; (11) CIMT plus Bimanual for children with CP to improve hand function; (12) Context Focused intervention for children with CP for functional motor task performance; (13) Ditto hand held devices for children with burns to provide procedural distraction and self‐management education; (14) Early Intervention using ABA for children with ASD; (15) Early Intervention using Developmental Care for preterm infants; (16) Family Centred Care for children with brain injury or CP, to improve children's function; (17) Parent education feeding intervention for children with disability to improve feeding competency and growth; (18) Physiological feeding intervention for children with disability; (19) Goal Directed Training for children with CP, to improve functional task performance; (20) Goal Directed Training for children with DCD, to improve functional task performance; (21) Handwriting Task‐Specific Practice for children with DCD; (22) Home Programs for children with CP, to improve functional task performance; (23) Home Programs for children with ID, to improve functional task performance; (24) Joint Attention for children with ASD to improve social interactions; (25) Mental Health interventions for children with ASD; (26) Mental Health interventions for children with developmental delay; (27) Mental Health interventions for children with mental health disorders; (28) Occupational therapy after botulinum toxin injections for children with CP to promote hand function; (29) Kinesiotape for children with CP to improve hand function; (30) Pain Management for children with chronic pain secondary to physical disability and or chronic health conditions; (31) Parent Education using mindfulness for parents of children with ASD to reduce parental stress; (32) Parent Education using problem solving for parents of children with ASD to reduce parental stress; (33) Parent Education for children with disabilities to promote parenting confidence; (34) Parent Education for children with behaviour disorders to improve parent well‐being; (35) PECS for children with ASD to promote communication; (36) Positioning in NICU for preterm infants to promote normal movement development; (37) Pressure Care for children with CP using mattresses and cushions; (38) Social Skills Training mediated by peers for children with ASD; (39) Treadmill training for children with Down Syndrome to accelerate the onset of independent walking; (40) Weight loss using a family education and activity program called ‘Mighty Moves’ for children with obesity.

**Figure 2:**
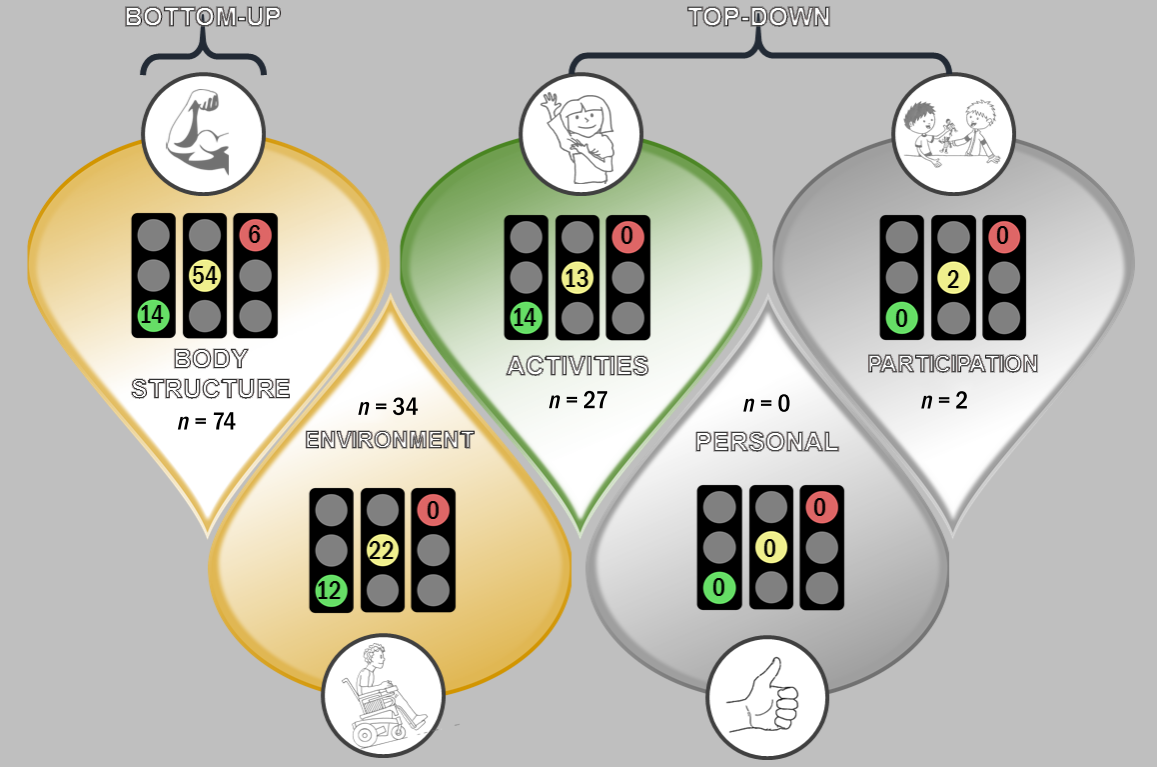
Occupational Therapy Interventions and the International Classification of Function

We assigned an ICF category to the primary and secondary intervention outcome of each intervention. Using the primary ICF level code, we mapped the profile of the paediatric OT evidence base to the ICF framework (Fig. 2). Green light effective interventions existed at the body structures and function ICF level (*n* = 14/74 indications (19%)), the activity level (*n* = 14/27 indications (52%)) and the environment level (*n* = 12/34 indications (35%)). When we compared the proportions of green light to yellow light to red light interventions by ICF levels, the activity level contained the largest number of green lights. At the activity level where there was 27 indications, green lights outweighed the number of yellow and red lights (Gree *n* = 14/27; Yellow = 13/27; Red = 0/27), meaning the most common traffic code at the activity level was green, which we illustrated by green shading in Figure 2. At the body structures and function ICF level, the most common traffic code was yellow, which we illustrated by yellow shading in Figure 2. All the red lights within the evidence base existed at the body structures and function level. At the environmental level, the most common traffic code was also yellow, which we illustrated by yellow shading in Figure 2. Two intervention's primary ICF code was at the participation level (Willis et al., 2010) and none at the personal level, indicating gaps in the occupational therapy evidence base, which we illustrated using grey shading in Figure 2. The two participation codes were weak positive, but these were based on trials that used activity‐based interventions and assumed an upstream participation gain, which was not well‐supported.

### **C**omparisons

In the included papers, consistent with conventional beliefs about it being unethical to withhold early intervention from children, rarely did researchers design studies where the control group received no intervention. In most studies, the controlled comparison was usual care. Some researchers carried out short duration studies using a wait‐list control design, where the control group received the experimental intervention after study completion.

CIMT for children with CP, was the only intervention comprehensively and empirically compared to other intervention options, using head‐to‐head RCT comparisons identified in our search strategy. CIMT was: (i) compared head‐to‐head with Bimanual Training showing no difference between the approaches (Sakzewski *et al*., 2015; Tervahauta, Girolami & Øberg, 2017); and (ii) combined with Bimanual Training and/or Botulinum toxin A, showing no additive benefits occurred from a combined intervention approach (Hoare *et al*., 2013). These researcher's concluded ‘intensity’ of practice was the key ingredient of these effective CP approaches (Sakzewski *et al*.; Tervahauta *et al*., 2017).

A meta‐analysis of intervention options for children with DCD compared the relative effect of DCD motor interventions by calculating and comparing effect sizes (Smits‐Engelsman *et al*., 2013). The authors calculated that ‘top‐down’ approaches (effect size = 0.89) were more effective than ‘bottom‐up’ approaches (effect size = 0.12).

**Figure 3:**
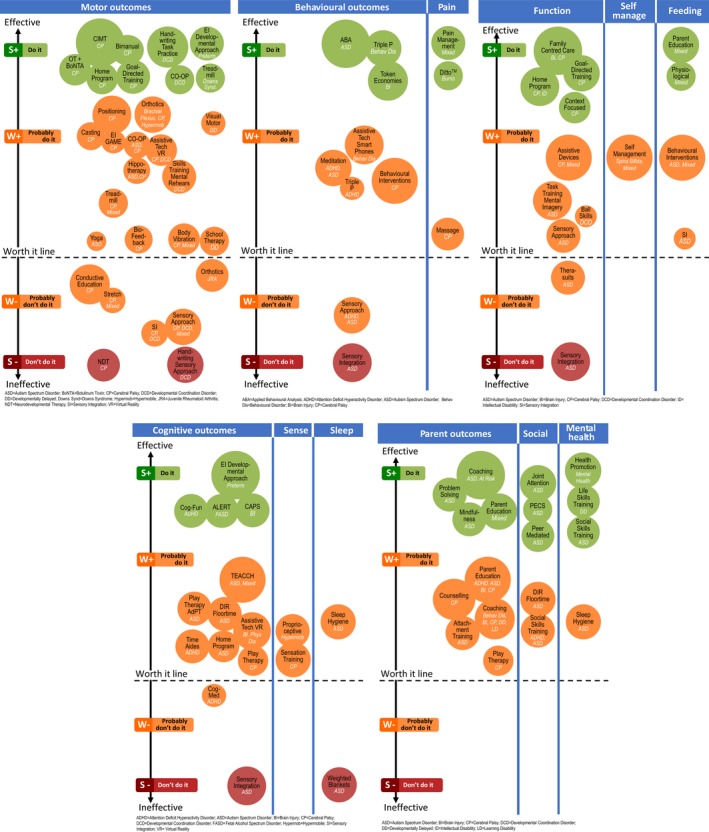
Bubble Charts Comparing the Effectiveness of Different Occupational Therapy Indications for Different Diagnoses

To assist with comparative clinical decision‐making across the paediatric occupational therapy evidence base, we created bubble charts. We mapped the 52 identified paediatric occupational therapy interventions, across 22 diagnoses, spanning 135 intervention indications, which sought to provide analogous outcomes, by diagnosis, into separate bubbles. In the bubble charts, the size of the bubble indicated the volume of published evidence, which was calculated by counting the number of published studies on the topic. The location of the bubble on the *y*‐axis of the graph corresponded to the GRADE system rating. The colour of the bubble denoted the Traffic Light Evidence Alert System rating (Fig. 3).

## Discussion

We set out to systematically summarise the current intervention options available to paediatric occupational therapists across different childhood disability populations. We found 40 interventions that received a ‘strong’ recommendation for use, indicating a high‐quality evidence base with more benefits than harms. These ‘green light’ interventions included: Behavioural Interventions (including ABA, Triple P and Token Economies); Bimanual; Coaching; Cognitive Cog‐Fun and CAPS; CO‐OP; CIMT; CIMT plus Bimanual; Context‐Focused; Ditto; Early Intervention (including ABA and Developmental Care); Family Centred Care; Feeding interventions (including coaching and physiologic); Goal Directed Training; Handwriting Task‐Specific Practice; Home Programs; Joint Attention; Mental Health Interventions; occupational therapy after BoNT; Kinesiotape; Pain Management; Parent Education; PECS; Positioning in NICU; Pressure Care; Social Skills Training Peer Mediated; Treadmill training and Weight loss ‘Mighty Moves’.

The paediatric occupational therapy evidence base is under immense growth and expansion. The SRs and trials greater than 10 years old were predominantly about CP with one study about Brachial Plexus and DD. Almost always these older studies showed that the ‘bottom‐up’ interventions were ineffective with no difference between the experimental and control groups.

### Clinical Implications

Occupational therapists working with children and their parents have several evidence based interventions to choose from. The strength of this paper is that it provides a systematic, clear and concise summary of all the available interventions by diagnosis with an easy to interpret summary of efficacy. There are some important learnings:

#### A. Parent partnership within occupational therapist intervention is effective and worthwhile

Occupational Therapists embrace the principles of family centred care (Hanna & Rodger, 2002) where the parent is the decision‐maker and the expert in knowing their child and the therapist is a technical resource to the family. We found that 13% of paediatric occupational therapy interventions are directed at the parent, so parents can deliver intervention at home within daily parenting. Evidence suggests that parent‐delivered intervention is equally effective to therapist‐delivered intervention (Baker *et al*., 2012), which is not surprising given parent's knowledge of their children's preferences and engagement style, and the volume of caregiving they carryout (Smith, Cheater & Bekker, 2015). In the diagnoses studied (ADHD, ASD, At risk, Behavioural Disorders, BI, CP, DD, LD, obesity), it was very clear that parents respond well to parent education and training (Antonini *et al*., 2014; Barlow, Smailagic, Huband, Roloff & Bennett, 2012; Case‐Smith & Arbesman, 2008; Dykens, Fisher, Taylor, Lambert & Miodrag, 2014; Feinberg *et al*., 2014; Hanna & Rodger, 2002; Howe & Wang, 2013; Kuhaneck, Madonna, Novak & Pearson, 2015; Lawler, Taylor & Shields, 2013; Tanner, Hand, O'toole & Lane, 2015; Zwi, Jones, Thorgaard, York & Dennis, 2011), consistent with family centred philosophy about parents’ aspirations of parenting well, to help their children (Hanna & Rodger, 2002). Moreover, parents and children carry out intervention effectively at home, and therefore home programs (Novak & Berry, 2014b; Novak *et al*., 2013; Sakzewski, Ziviani & Boyd, 2013; Sakzewski *et al*., 2015; Wuang, Ho & Su, 2013) and self‐management programs (Lindsay, Kingsnorth, Mcdougall & Keating, 2014; Moola, Faulkner, White & Kirsh, 2014) are an effective method for increasing the intensity of therapy.

When carrying out parent education, literature tells us that parents need and want: knowledge of the condition and intervention options; help accessing support services; and advice about coping strategies, via a collaborative partnership (Smith *et al*., 2015). Even though family centred practice has existed since the 1990s, parents still experience some resistance to their input from health professionals (Smith *et al*.). Unclear expectations about roles further elevate parental stress (Coyne, 2015). Occupational therapists therefore need to be mindful of parent's experiences and aim to clearly communicate information and coach parents to guide care, to optimise family outcomes (Coyne).

#### B. Activities‐based, ‘top‐down’ interventions deliver bigger gains

Numerous occupational therapy interventions exist, aiming to improve motor, behavioural and functional outcomes (Fig. 3), affording a lot of choice to families and clinicians. The greatest number of effective green light interventions was at the activity level of the ICF, indicating that daily life skills training using a ‘top‐down’ approach is a strength of the occupational therapy profession. Examples include: Bimanual Training; CIMT; CO‐OP; GAME; Goal‐Directed Training; Handwriting Task Training; Home Programs using Goal‐Directed Training; Social Skills Training; and Task Training. Consistent with current knowledge about the conditions for inducing neuroplasticity (Kleim & Jones, 2008), the green light, ‘top‐down’, activity level interventions all have the following key ingredients in common: (i) begin with the child's goal, to optimise motivation and saliency of practice; (ii) practice of real‐life activities in natural environments to optimise the child's learning and the variability of the practice; (iii) intense repetitions to activate plasticity, including home‐based practice; and (iv) scaffolded practice to the ‘just right challenge’ to enable success under self‐generated problem‐solving conditions, to optimise enjoyment.

In contrast, some of the most established paediatric occupational therapy interventions NDT/Bobath and SI were originally developed as ‘bottom‐up’ interventions. NDT/Bobath and SI originated in an era of medicine when intervention aimed to remediate the child's body structural deficits, thinking function would emerge (Rodger *et al*., 2005; Rodger *et al*., 2006). However, over time the NDT/Bobath and SI approaches have been broadened to also accommodate use of ‘top‐down’ functional training approaches. Fidelity to the original NDT/Bobath and SI approach therefore varies greatly (Mayston, 2016), and as such, a leading Bobath expert has recently stated that Bobath ‘no longer stands for a valid universal therapy approach’ (Mayston, 2016, p. 994). This means that interpreting the meaning of historical NDT/Bobath and SI research evidence about efficacy within the context of contemporaneous clinical practice is challenging. The efficacy of both NDT/Bobath and SI have been critiqued within SRs (Boyd & Hays, 2001; Brown & Burns, 2001; Case‐Smith & Arbesman, 2008; Case‐Smith, Clark & Schlabach, 2013; Case‐Smith, Weaver & Fristad, 2015; Lang *et al*., 2012; May‐Benson & Koomar, 2010; Novak *et al*., 2013; Sakzewski, Ziviani & Boyd, 2009; Sakzewski *et al*., 2013; Steultjens *et al*., 2004; Watling & Hauer, 2015; Weaver, 2015) and these data mostly relate to older trials. SR authors have concluded that NDT/Bobath and SI rarely confer motor gains superior to no intervention, but the RCTs contain so many methodological flaws that recommendations for use or discontinuation of use within practice cannot be made with certainty (Boyd & Hays, 2001; Brown & Burns, 2001; Case‐Smith & Arbesman, 2008; Case‐Smith *et al*., 2013; Case‐Smith *et al*., 2014; Lang *et al*., 2012; May‐Benson & Koomar, 2010; Novak *et al*., 2013; Sakzewski *et al*., 2009, 2013; Steultjens *et al*., 2004; Watling & Hauer, 2015; Weaver, 2015). Some therapists have interpreted the uncertainty of the NDT/Bobath and SI systematic evidence as justification of continuance, whereas others in the profession recommend discontinuance because of the growing body of ‘top‐down’ evidence that offer effective alternatives (Rodger *et al*., 2006). A Bobath expert has recommended that the common‐sense way forward for the profession is to choose interventions that promote activity and participation outcomes (Mayston, 2016) and to use consistent language to describe intervention options. For example, describing interventions by clear uniform terminology (i.e. ‘splitting’) might be more helpful than ‘clumping’ interventions into expanded NDT/Bobath umbrella terms.

We analysed the breakdown of the effectiveness of motor interventions, above and below the worth it line (Fig. 3), in terms of ‘bottom‐up’ vs. ‘top‐down’, and a trend favouring ‘top‐down’ emerged. Of the seven motor intervention indications below the ‘worth it line’, coded on GRADE as weak negative or strong negative (red), 7/7 (100%) were ‘bottom‐up’ approaches. Of the 22 motor intervention indications above the ‘worth it line’ eight were green and 14 were yellow: 8/8 (100%) green indications (strong positive) were ‘top‐down’. A similar trend emerged in the comparative effectiveness analysis of functional interventions. Of the seven functional intervention indications above the ‘worth it line’, coded on GRADE as strong positive (green), 4/4 (100%) were ‘top‐down’. There were a small number of studies using SI and the sensory approach to improve function coded on GRADE as weak positive, but the studies had a high risk of bias and SR authors recommended interpreting the positive results with caution (Case‐Smith *et al*., 2014; Case‐Smith *et al*., 2015; Watling & Hauer, 2015).

### Research Implications

The following areas of the evidence‐base would benefit from more research: (i) *Parent Education*: None of the parent education approaches were ineffective. Thus, more research is worthwhile exploring parent's preferred learning styles and levels of support required to manage the stress of raising a child with a disability. There are potential financial gains to the health system by thoroughly understanding effective parent interventions, because parent‐delivered intervention is equally effective and less expensive; (ii) *Head‐to‐head comparisons*: Head‐to‐head comparisons of different interventions aiming to achieve the same outcomes, in well‐controlled trials with cost‐effectiveness data, would enable determinations about best practice to be made from good evidence, and thus inform parent and policy‐maker's decision‐making; (iii) *‘Dose’ comparison studies*: ‘Dose’ comparison studies using well controlled intensity trials would enable occupational therapists to better inform parents about ‘how much’ intervention is enough; and (iv) *Participation Interventions*: There is a clear gap in the evidence‐base about interventions that directly improve a child's participation in life and should be the focus of future RCTs and other rigorous methodologies. CIMT, Bimanual and Home Program occupational therapy interventions were measured to confirm whether or not they conferred participation gains, and the clinical trials demonstrated no between group differences (Adair, Ullenhag, Keen, Granlund & Imms, 2015). These results indicate that there is a clear need to develop interventions that specifically target participation, rather than anticipating activities‐based interventions will confer upstream participation gains. Changes in participation are multifactorial and involve individual factors, contextual factors, the nature of the participation activity and the environment in which the activity is being performed (Imms *et al*., 2017). Any new participation intervention invented, will need to address all of these factors to be successful.

### Limitations

Our review has several limitations. First, we only included SRs and RCTs because we aimed to analyse best‐available evidence, but means some intervention approaches will have been excluded and overlooked because no trials or reviews existed. Second, this was an analysis of secondary data sources and reporting bias and publication bias may be in operation, because positive findings have a higher chance of being published. This evidence may exist suggesting some interventions are ineffective which we were unable to review. Third, our search terms included ‘occupational therapy’ and thus will have excluded other effective interventions used by occupational therapists, but not invented or published by occupational therapists e.g. ‘Triple P’ for children with CP. Fourth, our paper was designed to provide an overview for clinicians indicating which interventions are effective, however, it does not provide enough detail about any one intervention to guide administration or training in any specific intervention. Clinicians need to refer directly to the cited article and more widely in the published literature for this information. Our findings must be interpreted within the context of our study limitations.

## Conclusions

This review provides a high‐level summary of effective paediatric occupational therapy interventions. Thirty‐nine effective intervention indications exist, offering both families and clinicians many choices to match their preferences and expertise. The paediatric occupational therapy evidence base suggests a growing trend towards activities‐level, ‘top‐down’ approaches and parent education, over and above ‘bottom‐up’ approaches. There are important ethical implications of translating these effective evidence‐based occupational therapy intervention options into clinical practice to give children the best chance at achieving their goals.

## Key points for occupational therapy

- Collaboration with parents is effective and worthwhile.
- Activities‐based, top‐down interventions confer larger clinical gains, than bottom‐up approaches, when aiming to improve a child's function.

## Authorship

All authors declare that this is original work and that they meet the criteria for authorship. Iona Novak designed the study, extracted the data, conducted the analyses and wrote the manuscript. Ingrid Honan conducted the analyses and wrote the manuscript. All authors read and approved the final manuscript.

## Funding

The study was unfunded and there are no competing financial disclosures.

## Conflict of interest

The authors have no conflicts of interest to disclose.

## Supporting information


**Table S1.** Main results table.
