# Supplementary material for: Effectiveness of paediatric occupational therapy for children with disabilities: A systematic review
Source: Aust Occup Ther J. 2019 Apr 10;66(3):258–73. doi: 10.1111/1440-1630.12573 (PMC6850210; doi:10.1111/1440-1630.12573)
Supplement: Supplementary file 1 — Table S1. Main results table. [file AOT-66-258-s001.docx]

**Table 1: Included Studies**

| **WHAT Intervention?** | **Intervention outcome Indications** | **Population** | **WHO delivered?** | **WHERE?** | **HOW MUCH/ Intensity?** | **ICF Level [Primary, Secondary]** | **Outcome** | **Citation** | **Study Design** | **GRADE** | | **Traffic Light**  **Action** |
| --- | --- | --- | --- | --- | --- | --- | --- | --- | --- | --- | --- | --- |
|  |  |  |  |  |  |  |  |  |  | **Evidence Quality** | **Recommendation for Use** |  |
| **1. Acupuncture *[plus OT]*** | 1 | Cerebral Palsy | OT  PT | Clinic | Total = 60hrs | Body Structure | No difference between groups, no additive benefit of acupuncture | Duncan 2012 | RCT | Moderate | **Weak-** | **Yellow**  **MEASURE** |
| **2. Assistive Devices** | 2 | Arthrogyposis  Cerebral Palsy  Down Syndrome  Spina Bifida  *[AFOs, Orthotics, Standing Frames, Seating Systems, Wheelchairs]* | OT  PT | Clinic | Incalculable, insufficient data | Environment  Body Structure | Improved independence | Montero 2014 | SR of 27 studies  (9/27=RCTs) | Moderate | **Weak+** | **Yellow**  **MEASURE** |
|  | 3 | Cerebral Palsy *[Computer Access; Hoists, Powered Mobility; Sleep Positioning Systems; Standing Frames; Wheelchairs]* | OT | Clinic | Incalculable, insufficient data | Environment  Body Structure | Improved function  Improved independence & mobility  Enabled inclusive education  Improved weight bearing  Improved sleep positioning  Reduced caregiver burden | Novak 2013* | SR of 166 studies  Assistive Devices (13/15=SRs, 2/15=RCTs) | Very Low – Low | **Weak+** | **Yellow**  **MEASURE** |
|  |  | Cerebral Palsy | OT | Clinic | Total = 13.5-12816hrs | Environment  Body Structure | No improvements in function | Steultjens 2004* | SR of 17 studies  Devices (0/2=RCTs) | Low |  |  |
| **3. Assistive Technology** | 4 | Autism Spectrum Disorder  *[Computer, DVD, virtual reality]* | OT | School | Total = 15hrs (median). Range incalculable, insufficient data | Environment  Body Structure | Improved facial recognition  Improved communication  Improved literacy | Grynszpan 2014 | SR of 22 studies  (10/22=RCTs) | Moderate | **Weak+** | **Yellow**  **MEASURE** |
|  |  | *[Mobile and Tablet Technology]* | OT | School | Incalculable, insufficient data | Environment | Improved vocational skills | Weaver 2015* | SR of 23 studies  (0/6=RCTs) | Very Low |  |  |
|  | 5 | Behaviour Disorder *[Helping the Noncompliant Child (HNC) program by Smart Phone]* | Psychologist  OT | School | Total incalculable, insufficient data, but included videos, phone calls + SMS | Environment | Improved engagement  Improved behaviour | Jones 2014 | RCT | Low | **Weak+** | **Yellow**  **MEASURE** |
|  | 6 | Brain Injury  *[Virtual Reality]* | OT | School | Incalculable, insufficient data | Environment  Personal | VR systems are motivating but limited evidence to support improved motivation | Tatla 2014* | SR of 10 studies  VR (1/3=RCTs) | Very Low | **Weak-** | **Yellow**  **MEASURE** |
|  |  | Brain Injury *[Web Cam Coaching “MiTii”]* | OT  PT | Home | Total = 0-46hrs [Some children did not practice, indicating poor acceptability] | Body Structure  Environment | No between group differences for motor or visual perception or function | Sakzewski 2106 | RCT | Moderate |  |  |
|  | 7 | Cerebral Palsy  *[Virtual Reality]* | OT | School  Community | Total = 3-12hrs | Environment  Participation | Conflicting results | Bonnechere 2014 | SR of 31 studies  (7/31=RCTs) | Very Low | **Weak+** | **Yellow**  **MEASURE** |
|  |  | Cerebral Palsy  *[Virtual Reality]* | OT | Home  Clinic | Total = 6-25hrs | Environment  Body Structure | Improved upper limb function | Chen 2014a | SR of 14 studies  (3/14=RCTs) | Low |  |  |
|  |  | Cerebral Palsy - Hemiplegia  *[Virtual Reality, Wii]* | OT | Home  Clinic | Total = 18hrs | Environment Body Structure | Improved strength  No between group differences for coordination | Chiu 2014 | RCT | Moderate |  |  |
|  |  | Cerebral Palsy  *[Virtual Reality]* | OT | School  Community | Incalculable, insufficient data | Environment  Participation | Induced neuroplasticity | Inguaggiato 2013* | SR of 7 studies  VR (0/1=RCTs) | Very Low |  |  |
|  |  | Cerebral Palsy *[Web Cam Coaching “MiTii”]* | OT | Home | Total = 3-74hrs | Activity  Body Structure  Environment | Improved function  Improved hand function but not clinically meaningful  Improved goal achievement  No between group differences for executive function | James 2015 NOTE Piovesana 2017 is the same trial | RCT | High |  |  |
|  |  | Cerebral Palsy *[Virtual Reality]* | OT | School  Community | Incalculable, insufficient data | Environment  Participation | Improved function | Novak 2013* | SR of 166 studies  VR (5/5=SRs) | Very Low – Low |  |  |
|  |  | Cerebral Palsy  *[Virtual Reality]* | OT | School  Community | Total = 3-20hrs | Environment  Participation | Improved motor skills  Confliciting results in lower level evidence | Snider 2010 | SR of 13 studies  (1/13=RCTs) | Very Low |  |  |
|  |  | Cerebral Palsy  *[Virtual Reality]* | OT | School  Community | Total = 1-14hrs  Over 4wks | Environment  Participation | Limited evidence to support improved motivation | Tatla 2013 | SR of 8 studies  VR (3/8=RCTs) | Very Low – Low |  |  |
|  | 8 | Developmental Coordination Disorder  *[Virtual Reality Wiifit]* | OT | School | Total = 2hrs | Environment  Body Structure | Improved motor skills | Hammond 2014 | RCT | Moderate | **Weak+** | **Yellow**  **MEASURE** |
|  | 9 | Physical Disability *[Computer Access; Virtual Reality]* | OT | School  Community | Incalculable, insufficient data | Environment  Participation | Enabled inclusive education by providing tools for learning & demonstrating learning  Facilitated interaction & communication  Increased participation in play | Chantry & Dunford 2010 | SR of 27 studies (1/27=RCT) | Very Low –Low | **Weak+** | **Yellow**  **MEASURE** |
| **4. Behavioural Interventions** | 10 | Attention Deficit Hyperactivity Disorder  *[Triple P]* | OT  Psychologist | Clinic  Home | Incalculable, insufficient data | Environment  Body Structure | Improved behaviour  Improved parenting skills | Au 2014 | RCT | Low | **Weak+**  *Triple P* | **Yellow MEASURE** |
|  | 11 | Autism Spectrum Disorder  *[ABA + Discrete Training]* | OT  Psychologist | Clinic  Home | Total = 32-400hrs | Body Structure | Improved behaviour  Improved communication | Case-Smith 2008* | SR of 49 studies  Behavioural (4/10=SR; 2/10=RCTs) | Moderate | **Strong+**  *ABA* | **Green GO** |
|  | 12 | Autism Spectrum Disorder *[Behavioural Interventions]* | OT  Psychologist | Clinic  Home | Incalculable, insufficient data | Body Structure  Environment | Improved behaviour but no effect on parental stress | Kuhaneck 2015* | SR of 34 studies  Behaviour  (2/2=RCTs) | Moderate | **Weak+**  *Behavioural interrvention* | **Yellow MEASURE** |
|  |  | Autism Spectrum Disorder  *[Behavioural Interventions including Milieu Therapy, Functional Communication*  *Training, & Pivotal Response Training]* | OT | Home | Incalculable, insufficient data | Body Structure  Environment | Improved joint attention  Improved communication Improved requesting  Improved spontaneous verb use | Tanner 2015* | SR of 66 studies  Behavioural  (3/3=SR) | Moderate |  |  |
|  |  | Autism Spectrum Disorder  *[Behavioural Interventions including Kata Training, Self Management]* | OT | Clinic  Home | Incalculable, insufficient data | Body Structure  Environment | Reduced challenging behaviour  Reduced repetitive behaviour | Tanner 2015* | SR of 66 studies  Behavioural  (4/5=SRs; 1/5=RCT) | Moderate |  |  |
|  | 13 | Behaviour Disorders  *[Triple P]* | Psychology  OT | Preschool | Total = 8hrs parent education + 1.5hr phone coaching | Environment  Body Structure | Improved child behaviour  Improved parenting skills maintianed long-term | Heinrichs 2014 | RCT | High | **Strong+**  *Triple P* | **Green GO** |
|  | 14 | Brain Injury  *[Token Economies, Contracts]* | OT | School  Child’s Community | Incalculable, insufficient data | Body Structure  Personal | Improved prospective memory  Limited evidence to support improved motivation | Tatla 2014* | SR of 10 studies  Token Economies (5/5=RCTs) | Moderate | **Strong+**  *Token Economies Contracts for memory* | **Green GO** |
|  | 15 | Cerebral Palsy | Psychology  OT | Clinic  Groups  Home | Incalculable, insufficient data | Body Structure  Personal | Improved behaviour  Improved parenting skills | Novak 2013* | SR of 166 studies  (1/3=SR, 2/3=RCTs) | Very Low – Low | **Weak+** | **Yellow**  **MEASURE** |
| **5. Bimanual Training *(also known as HABIT and HABIT-ILE)*** | 16 | Cerebral Palsy, Hemiplegia | OT  PT | Clinic | Total = 90hrs | Activity  Body Structure | Improved upper limb & lower limb movement | Bleyenheuft 2014 | RCT | Moderate | **Strong+** | **Green GO** |
|  |  | Cerebral Palsy, Hemiplegia | OT  Clown | Camp  Home | Total = 30-60hrs | Activity  Body Structure | Improved hand efficiency but gains are small | Case-Smith 2013* | SR of 24 studies | Moderate |  |  |
|  |  | Cerebral Palsy, Hemiplegia | OT  Clown | Camp  Home | Total = 30-60hrs | Activity  Body Structure | Improved hand efficiency but gains are small | Novak 2013* | SR of 166 studies  Bimanual (1/3=SR, 2/3=RCT) | High |  |  |
|  |  | Cerebral Palsy, Hemiplegia | OT  Clown | Camp  Home | Total = 30-60hrs | Activity  Body Structure | Improved hand efficiency but gains are small | Sakzewski 2009* | SR of 19 studies Bimanual (1/1=RCTs) | Superseded by Sakzewski 2013 |  |  |
|  |  | Cerebral Palsy, Hemiplegia | OT  Clown | Camp  Home | Total = 30-60hrs in <6-weeks  >60hrs gives bigger gains | Activity  Body Structure | Improved hand efficiency but gains are small | Sakzewski 2013* | SR of 42 studies  Bimanual (1/1=RCTS) | Moderate |  |  |
|  |  | Cerebral Palsy, Hemiplegia | OT  PT | Clinic  Home | Total = 36hrs | Activity  Body Structure | No difference between groups for hand function  Low statistical power | Speth 2015 ^[78]^ | RCT  Bimanual v BoNTA v combination | Low |  |  |
| **6. Biofeedback** | 17 | Cerebral Palsy | OT | Clinic | Incalculable, insufficient data | Body Structure  Environment | Improved muscle, activation  Improved function Improved active range of motion | Novak 2013* | SR of 166 studies  Biofeedback (2/3=RCT) | Low | **Weak+** | **Yellow MEASURE** |
| **7. Coaching** | 18 | At Risk of Disability | OT  Parent  Psychologist | Clinic  Home | Incalculable, insufficient data | Environment  Activities | Improved educational outcomes | Novak 2014a | SR of 16 studies  (2/16=SRs; 7/16=RCTs) | High | **Strong+** | **Green GO** |
|  | 19 | Autism Spectrum Disorder  *[Parent Coaching of Development]* | Parents  OT | Home | Total = 12hrs coaching | Environment  Activities | Reduced parent stress | Estes 2014 | RCT | Moderate | **Strong+** | **Green GO** |
|  |  | Autism Spectrum Disorder | OT | Clinic  Home | Incalculable, insufficient data | Environment  Activities | Improved function | Weaver 2015* | SR of 23 studies  (0/1=RCTs) | Low |  |  |
|  |  | Autism Spectrum Disorder | OT  Parent  Psychologist | Clinic  Home | Incalculable, insufficient data | Environment  Activities | Improved educational outcomes | Novak 2014a | SR of 16 studies  (2/16=SRs; 7/16=RCTs) | Moderate |  |  |
|  |  | Autism Spectrum Disorder & Sensory Disorder | OT | Home | Total = 3-18hrs | Environment  Activities | Reduced parent stress  Improved behaviour | Miller-Kuhanek 2018 | SR of 4 studies  (3/4=RCTS) | Moderate |  |  |
|  | 20 | Behaviour Disorder | OT  Parent  Psychologist | Clinic  Home | Incalculable, insufficient data | Environment  Body Structure | Improved behaviour | Novak 2014a | SR of 16 studies  (2/16=SRs; 7/16=RCTs) | Very Low | **Weak+** | **Yellow MEASURE** |
|  | 21 | Brain Injury | OT  Psychologist | Clinic  Home | Incalculable, insufficient data | Environment  Body Structure | Improved parenting  Improved child behaviour | Mast 2014 | RCT | Low | **Weak+** | **Yellow MEASURE** |
|  | 22 | Cerebral Palsy | OT  Parent  Psychologist | Clinic  Home | Incalculable, insufficient data | Environment  Activities | Improved motor | Novak 2014a | SR of 16 studies  (2/16=SRs; 7/16=RCTs) | Moderate | **Weak+** | **Yellow MEASURE** |
|  | 23 | Developmental Disability | OT  Parent  Psychologist | Clinic  Home | Incalculable, insufficient data | Environment  Activities | Improved development  Improved motor  Improved communication | Novak 2014a | SR of 16 studies  (2/16=SRs; 7/16=RCTs) | Moderate | **Weak+** | **Yellow MEASURE** |
|  | 24 | Learning Difficulties | OT  Parent  Psychologist | Clinic  Home | Incalculable, insufficient data | Environment  Activities | Reduced parent stress | Novak 2014a | SR of 16 studies  (2/16=SRs; 7/16=RCTs) | Low | **Weak+** | **Yellow MEASURE** |
| **8. Cognitive Interventions** | 25 | Attention Deficit Hyperactivity Disorder *[*Cog-Fun*]* | OT | Clinic  Home | Total = 10hrs OT coaching at the clinic + home practice | Body Structures  Activities | Improved function  Improved inhibition  Improved working memory | Maeir 2014 | RCT | Moderate | **Strong+**  *Cog-Fun* | **Green GO** |
|  |  | Attention Deficit Hyperactivity Disorder *[*Cog-Fun*]* | OT | Clinic | Total = 12hrs | Body Structures  Activities | Improved function  Improved inhibition  Improved working memory | Hahn-Markowitz 2017 | RCT | High |  |  |
|  | 26 | Attention Deficit Hyperactivity Disorder *[Cogmed Working Memory Training (CWMT)]* | OT  Psych | Clinic  Home | Total = 12.5-19hrs | Body Structures  Activities | Improved verbal and non-verbal working memory storage but no generalisation to function | Chacko 2015 | RCT | Moderate | **Weak-**  *Cogmed* | **Yellow MEASURE** |
|  | 27 | Attention Deficit  Hyperactivity Disorder, Autism Spectrum Disorder; Cerebral Palsy; Intellectual Disability; Spina Bifida  *[Time Aides]* | OT | School | Total – 4.5-22hrs | Body Structures  Environment | Increased time-processing | Janeslätt 2014 | RCT | Moderate | **Weak+**  *Time Aides* | **Yellow MEASURE** |
|  | 28 | Brain Injury  *[Counsellor-Assisted Problem-Solving (CAPS)]* | OT | School | Total = 8hrs | Body Structure  Environment | Improved executive function long-term | Kurowski 2014 | RCT | High | **Strong+**  *CAPS* | **Green GO** |
|  | 29 | Fetal Alcohol Spectrum Disorder  *[ALERT program]* | OT | School | Total = 12hrs | Body Structure  Environment | Improved self-regulation | Pfeiffer 2018* | SR of 5 studies  ALERT (1/1=RCT) | Moderate | **Weak+** | **Yellow MEASURE** |
| **9. Cognitive Orientation to Occupational Performance**  **(CO-OP)** | 30 | Autism Spectrum Disorder | OT | Clinic  Home | Total = 10hrs | Activities | Improved function | Weaver 2015* | SR of 23 studies  (0/2=RCTs) | Very Low | **Weak+** | **Yellow MEASURE** |
|  | 31 | Cerebral Palsy | OT | Clinic | Total = 10hrs | Activities | Improved function  Better transference | Cameron 2017 | RCT (pilot) | Low | **Weak+** | **Yellow MEASURE** |
|  |  | Cerebral Palsy | OT | Clinic | Total = 10hrs | Activities | Improved goal achievement | Jackman 2018 | RCT (pilot) | Low |  |  |
|  | 32 | Developmental Coordination Disorder | OT | Clinic  Home | Total = 8.5-13hrs | Activities | Improved function | Armstrong 2012* | SR of 19 studies  CO-OP  (1/6=RCTs) | Low | **Strong+**  *Upgraded because delivers superior gains to bottom-up approaches* | **Green GO** |
|  |  | Developmental Coordination Disorder | OT | Clinic  Home | Incalculable, insufficient data | Activities | Improved goal achievement  Improved generalisation | Polatajko 2010* | SR of 20 studies  CO-OP (1/4=RCTs) | Low-Moderate |  |  |
|  |  | Developmental Coordination Disorder | OT | Clinic  Home | Total = 8.5-20hrs | Activities | Improved function | Smits-Engelsman 2013* | SR of 26 studies  CO-OP (2/26=SR, 1/3=RCTs) | Moderate |  |  |
| **10. Conductive Education** | 33 | Cerebral Palsy | Conductor  OT | School | Incalculable, insufficient data | Body Structure  Participation | Conflicting evidence  Majority of studies show no difference between the groups | Case-Smith 2013* | SR of 24 studies  (CE 1/2=RCTs) | Low | **Weak-** | **Yellow MEASURE** |
|  |  | Cerebral Palsy | Conductor  OT | School | Incalculable, insufficient data | Body Structure  Participation | Conflicting evidence  Majority of studies show no difference between the groups | Novak 2013* | SR of 166 studies  CE (2/2=SR) | Low |  |  |
| **11. Constraint Induced Movement Therapy (CIMT)** | 34 | Cerebral Palsy, Hemiplegia | OT  Parent  Clown | Clinic  Home  Camp | Total = 30-60hrs | Activity  Body Structure | Improved upper limb quality of movement and hand use | Case-Smith 2013* | SR of 24 studies  (CIMT 3/5=RCTs) | High | **Strong+**  Results equally effective in clinic, camp or home mode of delivery  Results equally effective for cast, mitt, sling, but children prefered removeable constraints | **Green GO** |
|  |  | Cerebral Palsy, Hemiplegia | OT  Parent | Home | Total = 28hrs | Activity  Body Structure | Improved upper limb quality of movement and hand use | Chen 2014b | RCT | High |  |  |
|  |  | Cerebral Palsy, Hemiplegia | OT  Parent | Clinic  Home  Camp | Total = 30-60hrs | Activity  Body Structure | Improved upper limb quality of movement and hand use | Chen 2014c | SR of 27 studies  (27/27=RCTs) | High |  |  |
|  |  | Cerebral Palsy, Hemiplegia [Comparing 2 types of constraint] | Parent | Home | Total = 42hrs | Activity  Body Structure | Improved upper limb quality of movement and hand use | Christmas 2017 | RCT | High |  |  |
|  |  | Cerebral Palsy, Hemiplegia | OT  Parent | Clinic  Home | Total = 30-60hrs | Activity  Body Structure | Improved upper limb quality of movement and hand use | Hoare 2007 | SR of 3 studies  (1/3=RCTs) | Superseded by Sakzewski 2013 |  |  |
|  |  | Cerebral Palsy, Hemiplegia | OT  Parent | Clinic  Home | Total = 30-60hrs | Activity  Body Structure | Improved upper limb quality of movement and hand use | Huang 2009 | SR of 23 studies  (2/2=SR; 2/21=RCTs) | Superseded by Sakzewski 2013 |  |  |
|  |  | Cerebral Palsy, Hemiplegia | OT  Parent  Clown | Clinic  Home  Camp | Total = 30-60hrs | Activity  Body Structure | Improved upper limb quality of movement and hand use | Novak 2013* | SR of 166 studies  CIMT (5/5=SRs) | High |  |  |
|  |  | Cerebral Palsy, Hemiplegia | OT  Parent  Clown | Clinic  Home  Camp | Total = 30-60hrs in <6-weeks  >60hrs gives better outcomes | Activity  Body Structure | Improved upper limb quality of movement and hand use | Sakzewski 2009* | SR of 19 studies CIMT (4/4=RCTs) | Superseded by Sakzewski 2013 |  |  |
|  |  | Cerebral Palsy, Hemiplegia | OT  Parent  Clown | Clinic  Home  Camp | Total = 30-60hrs in <6-weeks  >60hrs gives better outcomes | Activity  Body Structure | Improved upper limb quality of movement and hand use | Sakzewski 2013* | SR of 42 studies  CIMT (22/22=RCTs) | High |  |  |
|  |  | Cerebral Palsy, Hemiplegia | OT  Parent  Clown | Clinic  Home  Camp | Total = 30-45hrs | Activity  Body Structure | Improved upper limb quality of movement and hand use | Sakzewski 2015* | RCT of equal dose group vs home program | High |  |  |
|  |  | Cerebral Palsy, Hemiplegia | OT | Clinic | Incalculable, insufficient data | Activity  Body Structure | Induced neuroplasticity | Inguaggiato 2013* | SR of 7 studies  (CIMT 0/6=RCTs) | Low |  |  |
|  | 35 | Cerebral Palsy  *[CIMT + Electrical Simulation]* | OT | Clinic | Total = 30hrs CIMT + 2hrs ES | Activity  Body Structure | Improved active range of motion & hand use | Xu 2015 | RCT | Moderate | **Weak+**  *CIMT + ES* | **Yellow MEASURE** |
| **12. Constraint Induced Movement Therapy &/or Bimanual** | 36 | Cerebral Palsy, Hemiplegia | OT | Groups | Total = 24-90hrs | Activity  Body Structure | Improved hand function | Sakzewski 2013* | SR of 42 studies  CIMT + Bimanual (2/2=RCTS) | High | **Strong+**  *Both CIMT & Bimanual interventions are effective & produce equal gains in activity and body structure. Either can be used and families can choose*  **Weak+**  *Participation* | **Green GO** |
|  |  | Cerebral Palsy, Hemiplegia | OT  Parent  Clown | Groups | Total = 60hrs | Participation | No difference between groups but a trend towards increased participation in younger and female participants | Adair 2015* | SR of 7 studies  CIMT + Bimanual (1 RCT) | Moderate |  |  |
|  |  | Cerebral Palsy, Hemiplegia | OT | Camp  Clinic  Home | Total = 24-210hrs over 1-10weeks | Activity  Body Structure | Improved hand function | Tervahauta 2017 | SR of 9 studies (9/9=RCTS)  CIMT v Bimanual | Low-High |  |  |
| **13. Context Focused *[Adapting Task & Environment]*** | 37 | Cerebral Palsy | OT  PT | Home | Incalculable, insufficient data | Environment  Activity | Improved function  No difference between groups i.e. both effective | Case-Smith 2013* | SR of 24 studies  (Context 1/1=RCTs) | High | **Strong+**  *Upgraded since* *both Task & Envrionment Adaptaions as effective as treating the child* | **Green GO** |
|  |  | Cerebral Palsy | OT  PT | Home | Incalculable, insufficient data | Environment  Activity | Improved function  No difference between groups i.e. both effective | Novak 2013* | SR of 166 studies  Context (1/1=RCTs) | High |  |  |
| **14. Ditto^TM^**  ***[Hand held education & distraction device]*** | 38 | Burns | OT  Ditto device | Clinic  Hospital | Incalculable, insufficient data | Environment  Body Structure | Faster wound re-epithelialisation  Reduced pain | Brown 2014 | RCT | High | **Strong+** | **Green GO** |
| **15. Early Intervention** | 39 | Autism Spectrum Disorder  [*Developmental, Individual-difference*  *Relationship-based [DIR] or floor time]* | OT | Home | Incalculable, insufficient data | Body Structure Activity | Improved communication  Improved parent-child interactions  Conflicting results in some studies | Tanner 2015* | SR of 66 studies  Early Intervention  (2/3=SR) | Moderate | **Weak+**  *Develop-mental* | **Yellow MEASURE** |
|  | 40 | Autism Spectrum Disorder  *[ABA, Behavioural Interventions, Parent training]* | OT  PT  SP  Parents | Clinic  Home | Total = 4-1820hrs | Body Structure Activity | Improved behaviour  Improved adaptive functioning  Authors recommend: (a) a combination of a behvioural and developmental approaches; and (b) involving parents | Zwaigen-baum 2016 | SR of 24 studies  (24/24=RCTs) | High | **Strong+**  *ABA* | **Green GO** |
|  | 41 | Cerebral Palsy *[NDT]* | OT  PT | Clinic  Home | Incalculable, insufficient data | Body Structure | No difference between groups for NDT vs Control | Novak 2013* | SR of 166 studies  Context (4/4=SRs) | Moderate | **Weak-**  *NDT*  *Downgraded because effective alternative GAME exists* | **Yellow MEASURE** |
|  |  | Cerebral Palsy *[NDT]* | OT  PT | Clinic  Home | Incalculable, insufficient data | Body Structure | No difference between groups for NDT vs Control | Morgan 2016a | SR of 34 studies  NDT  (7/7=RCTs) | Low-Moderate |  |  |
|  | 42 | Cerebral Palsy *[GAME]* | OT  PT | Home | Total = 9hrs | Activity  Environment | Improved motor skills | Morgan 2015 | RCT | Low | **Weak+**  *GAME* | **Yellow MEASURE** |
|  |  | Cerebral Palsy *[GAME]* | OT  PT | Home | Total = 216hrs | Activity  Environment | Improved motor skills  Improved cognition | Morgan 2016b | RCT | Moderate |  |  |
|  | 43 | Preterm  *[Developmental interventions]* | OT  PT  Physicians  Nurses  Psychologist  Parent | NICU  Clinic  Home | Total = 8hrs-2years | Body Structure  Environment | Improved parent-child interactions  Improved motor functions but gains are small | Case-Smith 2013* | SR of 24 studies  (2/5=SR; 3/5=RCTs) | High | **Strong+**  *Small, short term gains for motor and cognition* | **Green GO** |
|  |  | Preterm  *[Developmental interventions, DIR floortime]* | OT  PT  Physicians  Nurses  Psychologist  Parent | NICU  Clinic  Home | Total = 8hrs-2years | Body Structure  Environment | Improved parental recgoniton of child cues  Improved cognitve outcomes short term | Frolek Clark 2013* | SR of 13 studies (12/13=RCTs) | High |  |  |
|  |  | Preterm  *[Developmental interventions]* | OT  PT  Physicians  Nurses  Psychologist  Parent | NICU  Clinic  Home | Total = 8hrs-2years | Body Structure  Environment | Improved cognitive & motor functions but gains are small | Park 2014 | SR of 16 studies (16/16=RCTs) | High |  |  |
|  |  | Preterm  *[Developmental interventions]* | OT  PT  Physicians  Nurses  Psychologist  Parent | NICU  Clinic  Home | Total = 8hrs-2years | Body Structure  Environment | Improved cognitive outcomes in short-term but little effects on motor skills | Spittle 2007 | SR of 16 studies (6/16=RCTs) | Superseded by Spittle 2012 |  |  |
|  |  | Preterm  *[Developmental interventions]* | OT  PT  Physicians  Nurses  Psychologist  Parent | NICU  Clinic  Home | Total = 8hrs-2years | Body Structure  Environment | Improved cognitive outcomes in infancy, but gains were small and not sustained at school age. Little evidence of improved motor skills | Spittle 2012 | SR of 21 studies (10/21=RCTs) | High |  |  |
|  | 44 | Physical Disability  *[Therapy; family support; parent education;*  *school advice]* | OT  PT  SLP  Nursing  Social Work  Dietetics  Parent | Home  Clinic  Pre-School  School | Total = 9.75-26hrs (over 4-6months) | Body Structure  Environment | Increased parent satisfaction  Improved parent knowledge  Improved child development  Partly met information needs | Ziviani 2010 | SR of 10 studies (0/10=RCTs) | Very Low | **Weak+** | **Yellow MEASURE** |
| **16. Electrical Stimulation *(+/- exercise & motor training)*** | 45 | Cerebral Palsy | OT  PT | Clinic | Incalculable, insufficient data | Body Structure | Improved strength  Augmented botulinum toxin | Novak 2013* | SR of 166 studies  ES (5/5=SRs) | Moderate | **Weak+** | **Yellow MEASURE** |
|  | 46 | Spina Bifida | OT  PT | Clinic | Total = 10-42hrs  (0.5-1hr/dy for 4-56wks) | Body Structure | Improved strength | Dagenais 2009 | SR of 6 studies (0/6=RCTs) | Low | **Weak+** | **Yellow MEASURE** |
| **17. Family Centred Care** | 47 | Brain Injury; Cerebral Palsy | Parent with coaching from OT | Home | Total = 1-10hrs/ wk ongoing | Environment | Improved child functional skills  Improved parent satisfaction | Baker 2012 | SR of 5 studies (2/5=RCTs) | High | **Strong+** | **Green GO** |
| **18. Feeding Interventions** | 48 | Autism Spectrum Disorder *[Behavioural Interventions]* | OT | Clinic  Home | Incalculable, insufficient data | Body Structure  Activity | Reduced food refusal  Improved food consumption | Weaver 2015* | SR of 23 studies  (0/4=RCTs) | Very Low | **Weak+**  *Behavioural Intervention* | **Yellow**  **MEASURE** |
|  | 49 | Cerebral Palsy; Developmental Disability; Preterm Infants; Physical Disability *[Behavioural Interventions]* | OT | Clinic | Incalculable, insufficient data | Body Structure  Activity | Improved variety of foods accepted, weight gain, caloric intake and self-feeding | Howe 2013* | SR of 34 studies Behavioural (2/7=RCTs) | Low-Moderate | **Weak+**  *Behavioural Interventions* | **Yellow MEASURE** |
|  | 50 | Cerebral Palsy; Developmental Disability; Preterm Infants; Physical Disability  *[Parent Education]* | OT | Home | Incalculable, insufficient data | Environment  Body Structure | Improved growth, feeding competence and parent–child interactions | Howe 2013* | SR of 34 studies Parent Education  (n=4/6=RCTs) | Moderate | **Strong+**  *Parent Education* | **Green GO** |
|  | 51 | Cerebral Palsy; Developmental Disability; Preterm Infants; Physical Disability *[Physiological Interventions]* | OT | NICU  Home | Incalculable, insufficient data | Body Structure | Improved physiology from skin-to-skin, decreased feeding time from non-nutritive sucking, improved swallow safety from positioning | Howe 2013* | SR of 34 studies  Physiological (n=12/21=RCTs) | Moderate | **Strong+**  *Physiological Interventions* | **Green GO** |
| **19. Goal Directed Training [*including* Task Specific Training, Functional Training, *Neuromotor Task Training (NTT) & Motor Imagery*]** | 52 | Autism Spectrum Disorder  *[Task Specific Training]* | OT | Clinic | Incalculable, insufficient data | Activity | Improved work skills | Weaver 2015* | SR of 23 studies  (1/2=SR) | Very Low | **Weak+** | **Yellow MEASURE** |
|  | 53 | Cerebral Palsy *[Goal Directed Training]* | OT  PT | Clinic | Total = 12hrs | Activity | Improved dexterity | Crompton 2007 | RCT | Low | **Strong+** | **Green GO** |
|  |  | Cerebral Palsy *[Goal Directed Training]* | OT  PT | Home | Incalculable, insufficient data | Activity | Improved hand function  Improved self-care  Improved gross motor | Novak 2013* | SR of 166 studies  GDT (4/5=RCTs) | High |  |  |
|  |  | Cerebral Palsy *[Goal Directed Training]* | OT | Home | Incalculable, insufficient data | Activity | No between group difference, improved function from either parent-set or child-set goals | Vroland-Nordstrand 2015 | RCT | Moderate |  |  |
|  | 54 | Developmental Coordination Disorder  *[Task Specific Training]* | OT | Clinic  Group  School | Incalculable, insufficient data | Activity | Improved function  Improved participation | Armstrong 2012* | SR of 19 studies  Task Specific (1/4=RCTs) | Low – Moderate | **Strong+**  *Upgraded because more effective than bottom-up approaches* | **Green GO** |
|  |  | Developmental Coordination Disorder  *[Task Specific Training]* | OT | Clinic  Group  School | Total = 4-30hrs | Activity | Improved handwriting | Smits-Engelsman 2013* | SR of 26 studies  Task Specific (2/26=SR, 3/13=RCTs) | Moderate – High |  |  |
|  | 55 | Learning Disability | OT  PT | School | Total = 20hrs | Activity | Improved ball skills from ball skill training | Westendorp 2014 | RCT | Moderate | **Weak+** | **Yellow MEASURE** |
| **20. Handwriting Interventions** | 56 | Developmental Coordination Disorder  *[Computer Instruction vs Sensiorimotor]* | OT | School | Total = 8hrs | Activity | Improved speed and fluency from computer based instruction for task specific paper practice over and above sensirimotor | Chang 2014 | RCT | Moderate | **Strong+**  *Task Training* | **Green GO** |
|  |  | Developmental Coordination Disorder  *[Task Practice & Motor Imagery]* | OT | Group  School  Home | Total = 8-56hrs  *Note: effective studies had a dose >20 sessions* | Activity | Improved speed  Improved legibility  Improved autonomy | Hoy 2011 | SR of 11 studies  Task Practice  (4/6=RCTs) | High |  |  |
|  | 57 | Developmental Coordination Disorder  *[Sensorimotor]* | OT | Clinic  School | Total = 6-17hr | Body Structure | No difference beteeen groups | Hoy 2011 | SR of 11 studies  Sensorimotor (2/2=RCTs) | High | **Strong-**  *Sensori-motor & Sesnory*  *Downgraded as less effective top-down approaches* | **Red STOP** |
|  |  | Developmental Coordination Disorder  *[Sensory-based without handwriting practice]* | OT | Clinic  School | Total = 6-10hrs | Body Structure | No difference beteeen groups  Some children got worse | Hoy 2011 | SR of 11 studies  Sensory Based (3/3=RCTs) | High |  |  |
| **21. Hippotherapy *[Therapeutic Horse Riding]*** | 58 | Autism Spectrum Disorder | OT | Riding Stables | Total = 12-18hrs | Environment | Improved social skills  Reduced symptoms | Pfeiffer 2018* | SR of 5 studies  Horse Riding (1/2=RCT) | Low – Moderate | **Weak+** | **Yellow MEASURE** |
|  | 59 | Cerebral Palsy | OT  PT | Riding Stables | Incalculable, insufficient data | Body Structure Activity | Improved gross motor  Improved postural control | Novak 2013* | SR of 166 studies  Hippotherapy (4/5=SR; 1/5=RCT) | Low – Moderate | **Weak+** | **Yellow MEASURE** |
|  |  | Cerebral Palsy | OT  PT | Riding Stables | Total = 6-7.5hrs | Body Structure Activity | Improved gross motor function | Whalen 2012 | SR of 9 studies  (2/9=RCTs) | Low – Moderate |  |  |
|  |  | Cerebral Palsy | OT  PT | Riding Stables | Total = 1-26hrs | Body Structure Activity | Improved postural control  Improved balance | Zadnkiar 2011 | SR of 10 studies  (3/10=RCTs) | Low – Moderate |  |  |
| **22. Home Programs** | 60 | Autism Spectrum Disorder | Parent coached by OT | Home | Incalculable, insufficient data | Activity  Environment | Improved cognition | Novak 2014b* | SR of 4 studies  Autism  (1/2=RCTs) | Low – Moderate | **Weak+** | **Yellow MEASURE** |
|  | 61 | Cerebral Palsy | Parent coached by OT | Home | Incalculable, insufficient data | Activity  Environment | Improved goal attainment  Improved function  Improved hand function | Novak 2014b* | SR of 4 studies  Cerebral Palsy  (2/2=RCTs) | High | **Strong+**  Activity and environment    **Weak+** Participation | **Green GO** |
|  |  | Cerebral Palsy | Parent coached by OT | Home | Total = 8hrs | Activity  Environment  Participation | Improved goal attainment  Improved function  Improved hand function  No evidence of improved participation | Novak 2013* | SR of 166 studies  Home Program (1/2=RCTs) | High |  |  |
|  |  | Cerebral Palsy | Parent coached by OT | Home | Total = 8hrs | Activity  Environment | Improved goal attainment  Improved function  Improved hand function | Sakzewski 2013* | SR of 42 studies  Home Program (1/1=RCTS) | High |  |  |
|  |  | Cerebral Palsy, Hemiplegia | Parent coached by OT | Home | Total = 45hrs | Activity  Environment | Improved hand function from either individual OT + home program to group-based CIMT and Bimanual | Sakzewski 2015 | RCT of equal dose group-based vs home program | High |  |  |
|  | 62 | Intellectual Disability | Parent coached by OT | Home | Total = 60hrs | Activity  Environment  Participation | Improved funciton  Improved fine motor  No evidence of improved participation | Wuang 2013  Also cited in Adair 2015* | RCT | High | **Strong+**  Activity  **Weak+**  Participation | **Green GO** |
| **23. Joint Attention** | 63 | Autism Spectrum Disorder | OT  Parent | Home  Preschool | Incalculable, insufficient data | Environment  Body Structures | Increased parent sensitivity to child cues  Increased congition | Frolek Clark 2013* | SR of 4 studies (3/4=RCTs) | High | **Strong+** | **Green GO** |
|  |  | Autism Spectrum Disorder  *[JASPER Joint Attention, Symbolic*  *Play Engagement & Regulation]* | OT | Clinic | Total = 12hrs inside 30hrs of ABA | Environment  Body Structures | No difference between groups but underpowered & both groups received ABA | Stickles Goods 2013 | RCT | Low |  |  |
|  |  | Autism Spectrum Disorder | OT | Home  School | Incalculable, insufficient data | Environment  Body Structures | Improved joint attention and imitation | Tanner 2015* | SR of 66 studies  Joint Attention  (3/3=RCTs) | High |  |  |
| **24. Massage**  ***[plus OT]*** | 64 | Autism Spectrum Disorder *[Qigong*  *Sensory Treatment Chinese medicine massage]* | Parent  OT | Home | Total = 20-30hrs daily parent-delivered massage | Body Structure | Decreased autism severity  Improved behavior  Improved communication  Improved sensory symptoms | Bodison 2018* | SR of 7 studies with 4 on Qigong Massage (3/4=RCTS) | Moderate | **Weak+** | **Yellow MEASURE** |
|  | 65 | Cerebral Palsy | OT  Swedish Massage | Clinic | Total = 18hrs | Body Structure | No differences between groups  No reduction in spasticity | Novak 2013* | SR of 166 studies  Massage  (3/3= RCTs) | Low | **Weak+**  *Upgraded as may reduce pain* | **Yellow MEASURE** |
| **25. Meditation / Mindfulness** | 66 | Attention Deficit Hyperactivity Disorder *[Mantra, Yoga, Relaxation Training]* | Yoga Instructor | Clinic  Home | Incalculable, insufficient data | Body Structure  Environment | No differences between groups  Little evidence to support or refute use | Krisanaprak-ornkit 2010 | SR of 4 studies  (4/4=RCTs) | Moderate | **Weak+** | **Yellow MEASURE** |
|  | 67 | Autism Spectrum Disorder  *[Mindfulness, Relaxation]* | Parent | Clinic  Home | Incalculable, insufficient data | Body Structure  Environment | Little evidence to support or refute use | Kuhaneck 2015* | SR of 34 studies  Mindfulness  (0/3=RCTs) | Moderate | **Weak+** | **Yellow MEASURE** |
| **26. Mental Health Interventions** | 68 | Autism Spectrum Disorder  *[Social Skills Programs]* | OT  Psychologist | Home  School  Community | Incalculable, insufficient data | Activity  Environment | Improved social behavior and self-management | Arbesman 2013 | SR of 124 studies (77/124=SRs & RCTs) | High | **Strong+** | **Green GO** |
|  | 69 | Developmental Disability  *[Life Skills Programs]* | OT  Psychologist | Home  School  Community | Incalculable, insufficient data | Activity  Environment | Improved social and life skills | Arbesman 2013 | SR of 124 studies (77/124=SRs & RCTs) | High | **Strong+** | **Green GO** |
|  | 70 | Mental Health Disorders  *[Health Promotion]* | OT  Psychologist | Home  School  Community | Incalculable, insufficient data | Activity  Environment | Better stress management | Arbesman 2013 | SR of 124 studies (77/124=SRs & RCTs) | High | **Strong+** | **Green GO** |
| **27. Neuro- Developmental Therapy (NDT)** | 71 | Cerebral Palsy | OT  PT | Clinic | Incalculable, insufficient data | Body Structure | No between group differences | Boyd 2001 | SR of 60 studies | Superseded by Sakzewski 2013 | **Strong-**  *Note down-graded because top down (CIMT, GDT) delivers better results* | **Red STOP** |
|  |  | Cerebral Palsy | OT  PT | Clinic | Incalculable, insufficient data | Body Structure | No between group differences  Inconsistent results  Some studies reported some benefits on sub-components while other studies did not | Brown 2001 | SR of 17 studies  (12/17= RCTs) | High |  |  |
|  |  | Cerebral Palsy | OT  PT | Clinic | Incalculable, insufficient data | Body Structure | No between group differences | Case-Smith 2013* | SR of 24 studies  (NDT 1=SR; NDT 6=RCTs) | High |  |  |
|  |  | Cerebral Palsy | OT  PT | Clinic | Incalculable, insufficient data | Body Structure | No between group differences | Novak 2013* | SR of 166 studies  NDT (3/3= SR) | High |  |  |
|  |  | Cerebral Palsy | OT  PT | Clinic | Incalculable, insufficient data | Body Structure | No between group differences | Sakzewski 2009* | SR of 19 studies NDT (2/2=RCTs) | Superseded by Sakzewski 2013 |  |  |
|  |  | Cerebral Palsy | OT  PT | Clinic | Incalculable, insufficient data | Body Structure | No between group differences | Sakzewski 2013* | SR of 42 studies  NDT (2/2=RCTs) | High |  |  |
|  |  | Cerebral Palsy | OT  PT | Clinic | Incalculable, insufficient data | Body Structure | No between group differences | Steultjens 2004* | SR of 17 studies  NDT (2/2=RCTs) | High |  |  |
| **28. Occupational Therapy after Botulinum Toxin A (BoNT A)** | 72 | Cerebral Palsy Non-ambulant | OT | Clinic  Home | Total = 4hrs + orthotics | Body Structure | Improved care and comfort | Copeland 2014 | RCT | High | **Strong+**  *A combination of BoNT & OT is more effective than OT alone in reducing impairment, improving activity level outcomes & goal achievement* | **Green GO** |
|  |  | Cerebral Palsy Hemiplegia | OT | Clinic  Home | Total = 4-156hrs + home programs | Activity  Body Structure | Improved goal attainment  Improved hand function | Fehlings 2010 | SR of 15 studies (9/15=RCTs) | High |  |  |
|  |  | Cerebral Palsy Hemiplegia | OT | Clinic  Home | Total = 13hrs | Activity  Body Structure | Improved hand function | Hoare 2004 | SR of 15 studies  (2/15=RCTs) | Superseded by Sakzewski 2013 |  |  |
|  |  | Cerebral Palsy | OT | Clinic  Home | Incalculable, insufficient data | Activity  Body Structure | Improved hand function | Hoare 2010 | SR of 10 studies  (10/10=RCTs) | High |  |  |
|  |  | Cerebral Palsy | OT | Clinic  Home | Total = 16hrs | Activity  Body Structure | No difference between groups  Improved goal attainment from CIMT + BoNT OR Bimanual + BoNT | Hoare 2013 | RCT | High |  |  |
|  |  | Cerebral Palsy | OT | Clinic  Home | Total = 9hrs | Activity  Body Structure | Improved hand function | Lannin 2006 | SR of 24 studies  (3/24=RCTs) | Superseded by Sakzewski 2013 |  |  |
|  |  | Cerebral Palsy | OT | Clinic  Home | Total = 8hrs + home program | Activity  Body Structure | Improved hand function | Lidman 2015 | RCT | Moderate |  |  |
|  |  | Cerebral Palsy | OT | Clinic  Home | Incalculable, insufficient data | Activity  Body Structure | Improved goal attainment  Improved hand function | Novak 2013* | SR of 166 studies  OT after BoNT (5/5=SR) | High |  |  |
|  |  | Cerebral Palsy | OT | Clinic  Home | Total = 2-156hrs | Activity  Body Structure | Improved hand function | Reeuwijk 2006 | SR of 12 studies  (3/12=RCTs) | Superseded by Sakzewski 2013 |  |  |
|  |  | Cerebral Palsy | OT | Clinic  Home | Total = 4-156hrs over 6 months | Activity  Body Structure | Improved goal attainment  Improved hand function | Sakzewski 2009* | SR of 19 studies OT after BoNT (7/7=RCTs) | High |  |  |
|  |  | Cerebral Palsy | OT | Clinic  Home | Total = 4-39hrs over 6 months | Activity  Body Structure | Improved goal attainment  Improved hand function | Sakzewski 2013* | SR of 42 studies  OT after BoNT (12/12=RCTs) | High |  |  |
| **29. Orthotics** | 73 | Brachial Plexus  *[+/- passive ROM]* | OT | Clinic | Incalculable, insufficient data | Body Structure  Environment | Improved upper limb function (17-100% gains) | Bialocerkowski 2005 | SR of 8 studies (0/8=RCTs) | Very Low- Low | **Weak+** | **Yellow MEASURE** |
|  | 74 | Brain Injury | OT | Clinic | Incalculable, insufficient data | Body Structure  Environment | Improved hand function but gains were not maintained when orthotic wearing stopped | Jackman 2014 | SR of 6 studies  Brain Injury  (0/0=RCTs) | Moderate | **Unknown**** | **Unknown**** |
|  | 75 | Burns  *[Microskin – digital skin scar cover up]* | Psychologist  OT | Hospital | Total = Daily for 8weeks | Body Structure  Environment | Improved socialisation  Improved cosmesis | Maskell 2014 | RCT | Moderate | **Weak+** | **Yellow MEASURE** |
|  | 76 | Cerebral Palsy  *[Functional Splints & Resting Splints]* | OT | Clinic | Incalculable, insufficient data | Body Structure  Environment | Improved hand function but gains were not maintained when orthotic wearing stopped | Jackman 2014 | SR of 6 studies  (6/6=RCTs) | Moderate | **Weak+**  *Functional Splints & Resting Splints* | **Yellow MEASURE** |
|  |  | Cerebral Palsy  *[Hand splints]* | OT  PT | Clinic | Incalculable, insufficient data | Body Structure  Environment | Improved upper limb function  Reduced contracture  Limited evidence to support or refute use | Novak 2013* | SR of 166 studies  Upper Limb (1/1=SR) | Very low |  |  |
|  |  | Cerebral Palsy  *[Hand Splints including Orthokinetic cuff, Thumb Opponens,*  *MacKinnon, Lycra Splints]* | OT | Clinic | Total = 1.5-2190hrs | Body Structure  Environment | Improved bilateral hand use  Improved hand function | Steultjens 2004* | SR of 17 studies  Hand splints (1/6=RCTs) | Low |  |  |
|  | 77 | Cerebral Palsy  *[Kinesio taping]* | OT  PT | Clinic | Total = 10,368hrs over 12wks | Body Structure  Environment | Improved muscle power  Improved independence | Kaya Kara 2015 | RCT | Moderate | **Strong+**  *Kinesio taping* | **Green GO** |
|  | 78 | Cerebral Palsy *[Casting]* | OT | Clinic | Total = 2-528hrs  Using 3-6 casts | Body Structure  Environment | Improved passive ROM short term  Limited evidence to support or refute use | Lannin 2007 | SR of 23 studies  (3/23=RCTs) | Low-Moderate | **Weak+**  *Casting* | **Yellow MEASURE** |
|  | 79 | Cerebral Palsy  *[Therasuits]* | OT  PT | Clinic | Incalculable, insufficient data | Body Structure  Environment | Conflicting evidence  Some studies show improved gross motor, other studies show no between group differences | Novak 2013* | SR of 166 studies  Therasuits (2/2=RCTs) | Low | **Weak-**  *Therasuits* | **Yellow MEASURE** |
|  |  |  | OT  PT | Clinic | Total = 6-36hrs | Body Structure  Environment | Improved stability but did not improve function | Wells 2017 | SR of 14 studies  Suits (5/14=RCTs) | Moderate |  |  |
|  | 80 | Joint Hypermobility Syndrome | OT  PT | Clinic | Incalculable, insufficient data – splint worn when handwriting | Body Structure  Environment | Increased handwriting speed while wearing the splint. No change in handwriting proficeincy | Smith 2014* | SR of 3 studies  Orthotics  (0/1=RCTs) | Low | **Weak+** | **Yellow MEASURE** |
|  | 81 | Juvenile Arthritis  *[Resting Splints]* | OT | Clinic | Total = 48hrs wrist rest in splint | Body Structure  Environment | No change in pain and stiffness from steriods plus upper limb wrist splinting and is therefore not recommended | Wallen & Gillies 2006 | SR of 7 studies (1/1=RCTs wrist; 5/6=RCTs knees) | Moderate | **Weak-**  *Resting Splints* | **Yellow MEASURE** |
| **30. Pain Management** | 82 | Chronic Pain from a range of diagnoses including physical disability & chronic health | Multidisciplinary including OT | Hospital | Total = 105-168hrs | Body Structure | Reduced pain  Reduced school absence  Reduced depression  Reduced catastrophizing  Reduced pain intensity and anxiety longer-term | Hechler 2014 | RCT | Moderate | Strong+  *Upgraded because of harm from not treating* | Green GO |
| **31. Parent Counselling** | 83 | Cerebral Palsy | OT | Clinic | Total = 1-13.5hrs | Environment | No difference between groups  *NOTE: More evidence in social work & psychology literature* | Steultjens 2004* | SR of 17 studies  Sensory-based (2/2=RCTs) | Moderate | **Weak+** | **Yellow MEASURE** |
| **32. Parent Education/ Parent Training** | 84 | Autism Spectrum Disorder  *[Parent Education]* | Parent coached by OT | Groups  Home  Clinic | Incalculable, insufficient data | Environment | Improved social skills  Reduced problem behaviours | Case-Smith 2008* | SR of 49 studies  Parent Training (0/3=RCTs) | Low | **Weak+**  *Parent Training* | **Yellow MEASURE** |
|  |  | Autism Spectrum Disorder  *[Parent Education]* | Parent coached by OT | Groups  Home  Clinic | Incalculable, insufficient data | Environment | Improved parent confidence  Confliciting results for reduced parental stress | Kuhaneck 2015* | SR of 34 studies  Parent Training  (6/11=RCTs) | Moderate |  |  |
|  |  | Autism Spectrum Disorder  *[Parent Education]* | Parent coached by OT | Groups  Home  Clinic | Incalculable, insufficient data | Environment | Improved communication  Improved joint attention | Tanner 2015* | SR of 66 studies  Parent Training  (2/2=RCTs) | Moderate |  |  |
|  | 85 | Autism Spectrum Disorder  *[Mindfulness]* | Parent coached by OT | Groups  Home  Clinic | Incalculable, insufficient data | Environment | *Mindfulness:*  Reduced parental stress and depression | Dykens 2014 | RCT | Moderate | **Strong+**  *Mindfulness* | **Green GO** |
|  | 86 | Autism Spectrum Disorder  *[Attachment Training, Cuing, Imitation]* | Parent  OT | Clinic  Home | Incalculable, insufficient data | Environment Body Structure | Improved parent-child attachment  Improved social-emaotional skills | Case-Smith 2008* | SR of 49 studies  Attachment Training (1/16=RCTs) | Low | **Weak+**  *Attachment Training* | **Yellow MEASURE** |
|  | 87 | Autism Spectrum Disorder  *[Problem Solving]* | Parent coached by OT | Groups  Home  Clinic | Incalculable, insufficient data | Environment | *Problem Solving Education:*  Reduced parental stress | Feinberg 2014 | RCT | Moderate | **Strong+**  *Problem Solving* | **Green GO** |
|  | 88 | Autism  Cerebral Palsy Developmental Delay  Speech Disorders  *[Parent Education]* | Parent coached by OT, SLP, PT or Social Worker | Home | Total = 0.5-2.5hrs per week ongoing | Environment | Caregivers/parents carried out interventions with equal efficacy to health professionals | Lawler 2013 | SR of 29 studies  (12/29=RCTs) (2/12=RCT delivered by OT) | High | **Strong+**  *Parent Training* | **Green GO** |
|  | 89 | Attention Deficit Hyperactivity Disorder  *[Parent Education]* | Parent | Parent Education Groups  Home | Total = 10-40hrs | Environment Body Structure | Improved behaviour  Reduced parental stress and improved confidence | Zwi 2011 | SR of 5 studies  (5/5=RCTs) | Moderate | **Weak+** | **Yellow MEASURE** |
|  | 90 | Behavioural Disorders [*Behavioural, Family Systems]* | Parent | Parent Education Groups and Clinic | Incalculable, insufficient data | Environment | Reduced parent depression, anxiety, stress, anger and guilt short-term  Improved parent confidence short-term | Barlow 2015 | SR of 48 studies  (48/48=RCTs) | High | **Strong+**  *Behavioural, Family Systems* | **Green GO** |
|  | 91 | Brain Injury  *[Parent Education]* | Parent | Online parent education | Total = 10-14hrs | Environment | Improved parenting skills  Improved child behaviour | Antonini 2014 | RCT | Low | **Weak+** | **Yellow MEASURE** |
|  | 92 | Cerebral Palsy  *[Parent Education]* | Parent | Parent Education Groups  Home | Incalculable, insufficient data | Environment Body Structure | Improved parenting skills to facilitate child development | Novak 2013* | SR of 166 studies  Parent Training (1/1=SR) | Very low | **Weak+** | **Yellow MEASURE** |
| **33. Picture Exchange**  **Communication System (PECS)** | 93 | Autism Spectrum Disorder | OT  SP | Clinic  School | Incalculable, insufficient data | Environment Activity | Improved social behaviour  Improved cooperative play  Improved joint attention  Improved requests and initiations | Tanner 2015* | SR of 66 studies  PECS  (2/6=SR; 1/6=RCTs) | Moderate | **Strong+** | **Green GO** |
| **34. Play Therapy** | 94 | Autism Spectrum Disorder [*DIR–floortime; pretend play; social stories]* | OT | Clinic  School | Incalculable, insufficient data | Activity  Participation | Improved pretend play  Improved adaptive behaviour | Tanner 2015* | SR of 66 studies  Play  (1/7=SR; 1/7=RCT) | Moderate | **Weak+**  *DIR* | **Yellow MEASURE** |
|  | 95 | Behaviour Disorders  *[Adlerian Play Therapy (AdPT)]* | Psychologist  OT | School | Total = 16hrs | Activity  Participation | Reduced disruptive behaviour | Meany-Walen 2014 | RCT | Moderate | **Weak+**  *AdPT* | **Yellow MEASURE** |
|  | 96 | Cerebral Palsy | OT | Clinic  School | Incalculable, insufficient data | Activity  Participation | Improved play skills  Improved child coping | Novak 2013* | SR of 166 studies  Play Therapy (1/1=RCT) | Low | **Weak+** | **Yellow MEASURE** |
| **35. Positioning** | 97 | Cerebral Palsy  *[Seating]* | OT | Clinic | Incalculable, insufficient data | Body Structure  Environment | Conflicting results – Some studies found improved hand function & posture  Other studies showed no between group differences | Stavness 2006 | SR of 16 studies (2/16=RCTs) | Low – Moderate | **Weak+**  *Seating* | **Yellow MEASURE** |
|  |  | Cerebral Palsy  *[Seating]* | OT | Clinic | Incalculable, insufficient data | Body Structure  Environment | Improved pulmonary function  Improved posture  Improved hand function | Novak 2013* | SR of 166 studies  Pressure Care (6/6=SR) | Very Low |  |  |
|  | 98 | Preterms  *[Dandle Roo containment]* | OT | NICU | Total = 462hrs (22hrs/day 3-4wks) | Body Structure  Environment | Less asymmetry of reflexes and movement | Madlinger-Lewis 2014 | RCT | High | **Strong+** | **Green GO** |
| **36. Pressure Care**  ***[Mattresses & Cushions]*** | 99 | Cerebral Palsy | OT | Clinic  Home | Incalculable, insufficient data | Body Structure  Environment | Reduced ulcer development | Novak 2013* | SR of 166 studies  Pressure Care (1/1=SR) | Low | **Strong+**  *Upgraded because of harms from not treating* | **Green GO** |
| **37. School Therapy** | 100 | Developmental Disability | OT  PT | School | Incalculable, insufficient data | Body Structure  Activity | No difference between groups for in-class versus out-of class | Cole 1989^]^ | RCT | Moderate | **Weak+** | **Yellow MEASURE** |
| **38. Self-Management** | 101 | Asthma  Cancer  Celiac  Chronic Illness  Congenital Heart  Craniofacial  Diabetes  Epilepsy  HIV  Inflammatory Bowel  *[Therapeutic Summer Recreation Camps]* | Nurse  Psychologists  Doctors  PT  OT | Camp | Total = 48-336hrs | Environment  Personal | Improved psychological wellbeing short-term  Improved social interactions  Improved acceptance  Improved quality of life | Moola 2014 | SR of 21 studies  (1/21=RCTs) | Moderate | **Weak+**  *Camps* | **Yellow MEASURE** |
|  | 102 | Spina Bifida  Juvenile Arthritis  *[Education]* | *Education =* OT  Psychologist  Nurse  *Homework =* Parent/Child | Clinic  Home | Total = 0.5-9hrs | Environment  Personal | Decreased family stress  Improved medication adherence  Decreased pain  Increased quality of life  Higher disease knowledge & responsibility taking | Lindsay 2014 | SR of 6 studies (2/6=RCTs) | Moderate | **Weak+**  *Education* | **Yellow MEASURE** |
| **39. Sensation Training** | 103 | Cerebral Palsy  *[Tactile]* | OT | Unknown | Unknown | Body Structure | Unknown no studies about tactile interventions | Auld 2014 | SR of 30 studies  (0/30=studies about children) | Unknown | **Weak+** | **Yellow MEASURE** |
|  |  | Cerebral Palsy  *[Somatosensory]* | OT | Clinic | Total = 18hrs | Body Structure | Improved goal attainment and function  No between group differences for sensation | McLean 2017 | RCT (pilot) | Low |  |  |
|  | 104 | Joint Hypermobility Syndrome  *[Proprioceptive Excercises]* | OT  PT | Clinic | Incalculable, insufficient data – splint worn when handwriting | Body Structure  Environment | Reduced pain  No between group differences for proprioceptive capabilities | Smith 2014* ^[122]^ | SR of 3 studies  Proprioceptive  (0/1=RCTs) | Low | **Weak+**  *Proprioceptive Excercises* | **Yellow MEASURE** |
| **40. Sensory Approach**  ***[brushing, therapy balls, weighted vests, warm-ups, sensory stimulation]*** | 105 | Attention Deficit Hyperactivity Disorder  *[Therapy Balls as Seats]* | OT | School | Total = 9months of school year, for seated classroom time | Body Structure  Environment | No difference between groups in terms of improved concentration and on task behaviour | Fedewa 2015 | RCT | Moderate | **Weak-**  *Behaviour* | **Yellow MEASURE** |
|  |  | Attention Deficit Hyperactivity Disorder  *[Weighted Vests]* | OT | School | Incalculable, insufficient data | Body Structure  Environment | Conflicting findings  Improved attention  Improved in seat behaviour  No difference between groups for impulse control and on-task behaviour | Bodison 2018* | SR of 7 studies Weighted vests (2/2=RCTs) | Moderate |  |  |
|  | 106 | Autism Spectrum Disorder | OT | Home  School | Total = daily within routine | Body Structure | No benefits observed on behaviour or function | Case-Smith 2015* | SR of 19 studies  Sensory-based (0/14=RCTs) | Low-Moderate | **Weak-**  *Behaviour* | **Yellow MEASURE** |
|  | 107 | Autism Spectrum Disorder [environment changes to tolerate dentistry] | OT | Clinic | Total = procedural time | Environment | Improved self-reported discomfort and pain  Improved participation in dentistry | Bodison 2018* | SR of 7 studies Environment Changes (1=RCTs) | Moderate | **Weak+**  *Comfort* | **Yellow MEASURE** |
|  | 108 | Autism Spectrum Disorder  *[Weighted blankets]* | OT | Home | Total = 12-16 nights of sleep | Body Structure  Environment | No between group differences for amount of sleep, time to fall asleep, or number of waking times | Gringras 2014 | RCT | Moderate | **Strong-**  *Sleep*  *Blankets well tolerated but downgraded due to safety concerns* | **Red STOP safety concerns** |
|  | 109 | Autism Spectrum Disorder | OT | Home  School | Incalculable, insufficient data | Body Structure | Conflicting results for social outcomes | Tanner 2015* | SR of 66 studies  Sensory-based  (2/2=SRs) | Low | **Weak+**  *Social* | **Yellow MEASURE** |
|  | 110 | Autism Spectrum Disorder | OT | Home  School | Incalculable, insufficient data | Body Structure | Improved engagement in multi-sensory activities  Improved motor skills  Conflicting results for behaviour | Watling 2015* | SR of 23 studies  Sensory-based (2/18=RCTs) | Low-Moderate | **Weak+**  *Goals*  Authors recommend interpreting results cautiously due to risk of bias | **Yellow MEASURE** |
|  | 111 | ADHD, Autism, Developmental Coordination Disorder, Learning Difficulty | OT | Camp  Clinic | Total = 8-45hrs | Body Structure | Inconclusive results  Some studies showed improved behaviour and motor skills but high risk of bias | Polatajko 2010* | SR of 20 studies Sensory-based (2/5=RCTs) | Low-Moderate | **Weak-**  *Motor*  *Downgraded as CO-OP or task specific achieves better outcomes* | **Yellow MEASURE** |
|  | 112 | Cerebral Palsy | OT | Clinic | Total = 10-54hrs | Body Structure | No difference between groups | Steultjens 2004* | SR of 17 studies  Sensory-based (1/2=RCTs) | Low-Moderate | **Weak-**  *Motor*  *Downgraded as GDT, CIMT & Bimanual achieves better outcomes* | **Yellow MEASURE** |
|  | 113 | Developmental Coordination Disorder | OT | Clinic | Incalculable, insufficient data | Body Structure | Small improvements in motor skills but high risk of bias | Armstrong 2012* | SR of 19 studies  SI (2/4=RCTs) | Moderate | **Weak-**  *Motor*  *Downgraded as CO-OP or task specific achieves better outcomes*  *When sensory (Effect size 0.12) compared to task-specific (Effect size 0.89), sensory was less effective* | **Yellow MEASURE** |
|  |  | Developmental Coordination Disorder | OT | Clinic | Total = 5-8hrs | Body Structure | Small benefits from kinaesthetic training | Smits-Engelsman 2013* | SR of 26 studies  Task Specific (2/26=SR, 1/3=RCTs) | Moderate |  |  |
| **41. Sensory Integration *[sensory diet, swinging, brushing, therapy balls, weighted vests, body socks]*** | 114 | Autism Spectrum Disorder | OT | Clinic | Incalculable, insufficient data | Body Structure | May have benefits but insufficient research | Case-Smith 2008* | SR of 49 studies  SI (8/8=SRs) | Superseded by Lang 2012 & Case-Smith 2015 | **Weak+**  *Goals*  *Authors recommend interpreting results cautiously due to risk of bias* | **Yellow MEASURE** |
|  |  | Autism Spectrum Disorder | OT | Clinic | Total = 10-81hrs | Body Structure | 2 studies showed improved goal attainment, but high levels of bias | Case-Smith 2015* | SR of 19 studies  SI (2/5=RCTs) | Moderate |  |  |
|  |  | Autism Spectrum Disorder | OT | School | Incalculable, insufficient data | Body Structure | Improved goal achievement and participation | Schaaf 2018 | SR of5 studies  SI (3/5=RCTs) | Moderate |  |  |
|  |  | Autism Spectrum Disorder | OT | Clinic | Total = 16-30hrs | Body Structure | Improved goal achievement  Improved sleep  Decreased stereotypy  Reduced caregiver burden | Watling 2015* | SR of 23 studies  SI (3/4=RCTs) | Moderate |  |  |
|  |  | Autism Spectrum Disorder | OT | Clinic | Total = 16-30hrs | Body Structure | Improved goal achievement | Weaver 2015* | SR of 23 studies  (1/1=RCTs) | Moderate |  |  |
|  | 115 | Autism Spectrum Disorder | OT | Clinic  School  Home  Camp | Incalculable, insufficient data | Body Structure | 14 studies found no benefit on behaviour & 5 worsened stereotypy & problem  behaviors  8 studies showed mixed results  3 studies suggested improvements but had high risk of bias | Lang 2012 | SR of 25 studies  (0/25=RCTs) | Low-Moderate *Some high quality observational studies* | **Strong-***Behaviour*  SI had no consistent positive effect & cannot be recommended *Downgraded as behavioural approaches (ABA) achieves better outcomes* | **Red STOP** |
|  |  | Autism Spectrum Disorder [weighted vests] | OT | Home  School | Incalculable, insufficient data | Body Structure  Environment | No benefits observed in attention or in-seat behaviour | Bodison 2018* | SR of 7 studies Weighted vests (0=RCTs) | Very Low |  |  |
|  |  | Autism Spectrum Disorder  *[Swinging]* | OT | School | Total = 0.1hrs  (2x5mins swinging) | Body Structure | No difference between groups for on-task behaviour, stereotypy & in-seat behaviour | Bodison 2018* | SR of 7 studies Swinging (1/1=RCTs) | Moderate |  |  |
|  | 116 | Cerebral Palsy | OT | Clinic | Incalculable, insufficient data | Body Structure | Conflicting results  Most studies show no benefits | Novak 2013* | SR of 166 studies  SI (1/1=SR) | Low | **Strong-**  *Motor*  *Downgraded as GDT, CIMT & Bimanual achieves better outcomes* | **Red STOP** |
|  | 117 | Developmental Coordination Disorder /Learning Difficulties | OT | Clinic | Total = 13-62.5hrs | Body Structure | Conflicting results  Some studies showed improved behaviour, attention and motor skills, but high risk of bias | May-Benson 2010 | SR of 27 studies  (13/27=RCTs) | Moderate-High | **Weak-**  *Motor*  *Downgradedas COOP achieves better outcomes* | **Yellow MEASURE** |
| **42. Skills Training via Mental Imagery** | 118 | Cerebral Palsy | OT | Clinic | Total = 20hrs  *NOTE: Low dose, therefore interpret cautiously* | Activities  Body Structure | No functional gains measures  No difference between groups when compared to sensory | Steultjens 2004* | SR of 17 studies  Skills Training (1/2=RCTs) | Low-Moderate | **Weak+**  *Mental Imagery* | **Yellow MEASURE** |
|  | 119 | Developmental Coordination Disorder | OT | Clinic  Groups | Incalculable, insufficient data | Activities  Body Structure | Improved motor skills  Skills training has a larger effect than general therapy and/or Sensory Integration | Polatajko 2010* | SR of 20 studies Skills Training  (1/3 SR, 2/3=RCTs) | Moderate | **Weak+**  *Mental Imagery* | **Yellow MEASURE** |
| **43. Sleep Interventions** | 120 | Autism Spectrum Disorder  *[Sleep Hygiene]* | OT  Parent | Home | Total = 4hrs parent education | Body Structure  Environment | Improved sleep activation | Malow 2014 | RCT | Moderate | **Weak+**  *Sleep* | **Yellow MEASURE** |
| **44. Social Skills Training**  ***[Social Skills Training, Behavioural Interventions,***  ***Psychosocial Treatments]*** | 121 | Attention Deficit Hyperactivity Disorder  *[Social skills training]* | Psychologist  OT for child training + Parent for Parent Training | School | Total = 8-104hrs | Activities  Participation | No differenes between groups  Trials had high risk of bias  Limited evidence to support or refute use | Storebo 2011 | SR of 26 studies  (11/26=RCTS) | Moderate | **Weak+**  *Social Skills Training* | **Yellow MEASURE** |
|  | 122 | Autism Spectrum Disorder *[Social skills training groups; peer mediatied]* | Parent  OT  Multidisciplinary team | School  Groups | Incalculable, insufficient data | Activities  Participation | Improved social skills  Improved confidence  Improved self esteem | Case-Smith 2008* | SR of 49 studies  Social Skills (0/8=RCTs) | Low  Superseded by Kasari, 2016 and Kamps, 2015 | **Strong+**  *Peer Training* | **Green GO** |
|  |  | Autism Spectrum Disorder *[Social Skills Training Groups; Peer Mediatied, Social Stories]* | Parent  OT  Multidisciplinary team | School  Groups | Incalculable, insufficient data | Activities  Participation | Improved social skills  Decreased solitary play  Conflicting results for peer mediated interteventions and social stories | Tanner 2015* | SR of 66 studies  Social Skills  (4/35=RCTs) | Low – Moderate  Superseded by Kasari, 2016 and Kamps, 2015 |  |  |
|  |  | Autism Spectrum Disorder *[Social Skills Training Groups]* | Parent  OT  Multidisciplinary team | School  Groups | Total = 8-12hrs | Activities  Participation | No differences between groups for peer networking  Increased peer engagement from skills training | Kasari 2016 | RCT | High |  |  |
|  |  | Autism Spectrum Disorder *[Peer Networking Training]* | Parent  OT  Multidisciplinary team | School  Groups | Incalculable, insufficient data | Activities  Participation | Improved peer interactions | Kamps 2015 | RCT | High |  |  |
| **45. Stretching *[passive: self-administered, therapist-administered and device-***  ***administered]*** | 123 | Brain Injury, Cerebral Palsy Charcot-  Marie-Tooth Duchenne Muscular Dystrophy  Stroke,  Spinal Cord Injury | OT  PT | Clinic  Home | Total = 0.25 -1512 hours | Body Structure | Stretch does not have clinically important effects on joint mobility in adults with or at risk of contractures.  Little evidence to support or refute use in cerebral palsy | Katalinic 2010 | SR of 35 studies  (35/35=RCTs) | High | **Strong-**  *Adults* | **Red STOP** |
|  | 124 | Cerebral Palsy | OT  PT | Clinic  Home | Incalculable, insufficient data | Body Structure | Little evidence to support or refute use in cerebral palsy | Novak 2013* | SR of 166 studies  SI (2/2=SR) | Moderate | **Weak-** | **Yellow MEASURE** |
| **46. Treatment and Education of Autistic and Communication**  **Handicapped Children (TEACCH)** | 125 | Autism Spectrum Disorder | Parent coached by OT & Teacher | School | Incalculable, insufficient data | Body Structure  Environment | Improved motor skills  Improved behaviour | Case-Smith 2008* | SR of 49 studies  TEACCH (0/5=RCTs) | Low | **Weak+** | **Yellow MEASURE** |
|  |  | Autism Spectrum Disorder | Parent coached by OT & Teacher | School | Incalculable, insufficient data | Body Structure  Environment | Conflicting results for social skills | Tanner 2015* | SR of 66 studies  TEACCH  (1/1=SR) | Low |  |  |
| **47. Therapeutic Listening/ Music Therapy** | 126 | Autism Spectrum Disorder  *[Music Therapy]* | OT  Music Therapist | Home  School | Total = 20-50hrs | Body Structure  Environment | Increased socially acceptable behaviour  Increased social responses  Increased verbal communication  Increased recognition of emotions  Decreased anxiety | De Vries 2015 | SR of 25 studies | Very Low | **Weak+** | **Yellow MEASURE** |
|  | 127 | Attention Deficit  Hyperactivity Disorder, Autism Spectrum Disorder, Arthritis, Cerebral Palsy, Down syndrome, Fibromyalgia, Learning Disabilities,  Rhett Syndrome, Stroke  *[The Listening Program (TLP)]* | OT | Clinic | Incalculable, insufficient data | Body Structure  Environment | Improved listening  Improved auditory processing  Improved language  High levels of bias from methodologies used, unpublished data, and the research was conducted by the commercial supplier of the program | Vargus 2015 | SR of 9 studies  (0/9=RCTs) | Low | **Weak+** | **Yellow MEASURE** |
| **48. Treadmill Training** | 128 | Down Sydnrome | OT  PT | Clinic  Home | Total = 9hrs | Body Structure  Activity | Improved strength  Improved agility | Lin 2012 | RCT | High | **Strong+** | **Green GO** |
|  | 129 | Cerebral Palsy | OT  PT | Clinic  Home | Incalculable, insufficient data | Body Structure  Activity | Improved walking | Novak 2013* | SR of 166 studies  Treadmill (4/4=SR) | Low | **Weak+** | **Yellow MEASURE** |
|  | 130 | Cerebral Palsy  Down Sydnrome  Spinal Cord Injury  Physical Disability | OT  PT | Clinic  Home | Incalculable, insufficient data | Body Structure  Activity | Improved walking speed but conflicting findings between studies  No adverse events | Zwicker 2010 | SR of 5 studies  (5/5=SRs) | Very Low – Low | **Weak+** | **Yellow MEASURE** |
| **49. Visual Motor Interventions *[Art & Craft]*** | 131 | Developmental Disability | OT | Clinic  School | Total = 8-52hrs | Body Structure  Activity | Improved fine motor skills  Improved visual motor skills | Case-Smith 2013* | SR of 24 studies  Visual Motor (0/4=RCTs) | Low | **Weak+** | **Yellow MEASURE** |
| **50. Weight Loss** | 132 | Childhood Cancer with obesity  *[Fit4Life – web and phone food and activity advice]* | Multidisciplinary including OT | Home | Total = 8hr phone counselling + web readings + SMS reminders | Body Structure Activity | Weight loss  Improved mood  No difference between groups for interpersonal problems and self-esteem | Huang 2014 | RCT | Moderate | **Weak+** | **Yellow MEASURE** |
|  | 133 | Childhood Obesity  *[Food Friends & Mighty Moves*, *Diet education & Parent-Led Home Activity Program]* | OT  Parent | Clinic  Home | Total = 18hrs education + 112hrs physical activity at home | Body Structure Activity | Improved physical activity | Bellows 2011 | RCT | High | **Strong+** | **Green GO** |
| **51. Whole Body Vibration** | 134 | Cerebral Palsy | PT  OT | Clinic | Incalculable, insufficient data | Body Structure | Improved strenght | Novak 2013* | SR of 166 studies  Whole Body Vibration (1/1=SR) | Low | **Weak+** | **Yellow MEASURE** |
|  | 135 | Physical Disability | PT  OT | Clinic | Total = 26hrs | Body Structure | Improved fitness | Matute-Llorente 2014 | SR of 22 studies  (8/22=RCTs) | Moderate | **Weak+** | **Yellow MEASURE** |
| **52. Yoga** | 136 | Autism Spectrum Disorder | OT | Clinic | Incalculable, insufficient data | Body Structure | Improved behaviour | Weaver 2015* | SR of 23 studies  (1/1=RCTs) | Moderate | **Weak+** | **Yellow MEASURE** |

*= cited more than once in this table for different interventions; **= not included in analyses as outcome unknown. ABA = Applied Behavioural Analysis; ADHD = Attention Deficit Hyperactivity Disorder; BoNT = Botulinum Neurotoxin; CE = Conductive Education; GDT= Goal Directed Training; hrs = Hours; NDT = Neuro-Developmental Therapy; OT = Occupational Therapist; PT = Physiotherapist; RCT = Randomised Controlled Trial; ROM = Range of Motion; SLP= Speech Language Pathologist; SMS = Short Message Service; SI = Sensory Integration; SP = Sensory Processing; SR = Systematic Review; VR = Virtual Reality; wks = Weeks

# REFERENCES

Arbesman, M., Bazyk, S., & Nochajski, S. M. (2013). Systematic review of occupational therapy and mental health promotion, prevention, and intervention for children and youth. *American Journal of Occupational Therapy, 67*(6), e120-e130. doi: 10.5014/ajot.2013.008359

Armstrong, D. (2012). Examining the evidence for interventions with children with developmental coordination disorder. *British Journal of Occupational Therapy, 75*(12), 532-540. doi: 10.4276/030802212X13548955545413

Au, A., Lau, K. M., Wong, A. H. C., Lam, C., Leung, C., Lau, J., & Lee, Y. K. (2014). The efficacy of a group Triple P (Positive Parenting Program) for Chinese parents with a child diagnosed with ADHD in Hong Kong: A pilot randomised controlled study. *Australian Psychologist, 49*(3), 151-162. [doi: 10.1111/ap.12053](https://doi.org/10.1111/ap.12053)

Auld, M. L., Russo, R., Moseley, G. L., & Johnston, L. M. (2014). Determination of interventions for upper extremity tactile impairment in children with cerebral palsy: A systematic review. *Developmental Medicine & Child Neurology, 56*(9), 815-832. [doi: 10.1111/dmcn.12439](https://doi.org/10.1111/dmcn.12439)

Bellows, L., Silvernail, S., Caldwell, L., Bryant, A., Kennedy, C., Davies, P., & Anderson, J. (2011). Parental perception on the efficacy of a physical activity program for preschoolers. *Journal of Community Health, 36*(2), 231-237. [doi: 10.1007/s10900-010-9302-1](https://doi.org/10.1007/s10900-010-9302-1)

Bialocerkowski, A., Kurlowicz, K., Vladusic, S., & Grimmer, K. (2005). Effectiveness of primary conservative management for infants with obstetric brachial plexus palsy. *International Journal of Evidence‐Based Healthcare, 3*(2), 27-44. [doi: 10.1111/j.1479-6988.2005.00020.x](https://doi.org/10.1111/j.1479-6988.2005.00020.x)

Bleyenheuft, Y., Arnould, C., Brandao, M. B., Bleyenheuft, C., & Gordon, A. M. (2015). Hand and Arm Bimanual Intensive Therapy Including Lower Extremity (HABIT-ILE) in children with unilateral spastic cerebral palsy: A randomized trial. *Neurorehabilitation and Neural Repair, 29*(7), 645-657. [doi: 10.1177/1545968314562109](https://doi.org/10.1177%2F1545968314562109)

Bodison, S. C., & Parham, L. D. (2018). Specific sensory techniques and sensory environmental modifications for children and youth with sensory integration difficulties: A systematic review. *American Journal of Occupational Therapy, 72*(1), 7201190040p1-7201190040p11. [doi: 10.5014/ajot.2018.029413](https://doi.org/10.5014/ajot.2018.029413)

Bonnechere, B., Jansen, B., Omelina, L., Degelaen, M., Wermenbol, V., Rooze, M., & Jan, S. V. S. (2014). Can serious games be incorporated with conventional treatment of children with cerebral palsy? A review. *Research in Developmental Disabilities, 35*(8), 1899-1913. [doi: 10.1016/j.ridd.2014.04.016](https://doi.org/10.1016/j.ridd.2014.04.016)

Brown, N. J., Kimble, R. M., Rodger, S., Ware, R. S., & Cuttle, L. (2014). Play and heal: Randomized controlled trial of Ditto™ intervention efficacy on improving re-epithelialization in pediatric burns. *Burns, 40*(2), 204-213. [doi: 10.1016/j.burns.2013.11.024](https://doi.org/10.1016/j.burns.2013.11.024)

Cameron, D., Craig, T., Edwards, B., Missiuna, C., Schwellnus, H., & Polatajko, H. J. (2017). Cognitive Orientation to daily Occupational Performance (CO-OP): A new approach for children with cerebral palsy. *Physical & Occupational Therapy in Pediatrics, 37*(2), 183-198. [doi: 10.1080/01942638.2016.1185500](https://doi.org/10.1080/01942638.2016.1185500)

Chacko, A., Bedard, A. C., Marks, D. J., Feirsen, N., Uderman, J. Z., Chimiklis, A., ... & Ramon, M. (2014). A randomized clinical trial of Cogmed Working Memory Training in school‐age children with ADHD: A replication in a diverse sample using a control condition. *Journal of Child Psychology and Psychiatry, 55*(3), 247-255. [doi: 10.1111/jcpp.12146](https://doi.org/10.1111/jcpp.12146)

Chang, S. H., & Yu, N. Y. (2014). The effect of computer-assisted therapeutic practice for children with handwriting deficit: A comparison with the effect of the traditional sensorimotor approach. *Research in Developmental Disabilities, 35*(7), 1648-1657. [doi: 10.1016/j.ridd.2014.03.024](https://doi.org/10.1016/j.ridd.2014.03.024)

Chantry, J., & Dunford, C. (2010). How do computer assistive technologies enhance participation in childhood occupations for children with multiple and complex disabilities? A review of the current literature. *British Journal of Occupational Therapy, 73*(8), 351-365. [doi: 10.4276%2F030802210X12813483277107](https://doi.org/10.4276%2F030802210X12813483277107)

Chen, Y. P., Lee, S. Y., & Howard, A. M. (2014a). Effect of virtual reality on upper extremity function in children with cerebral palsy: A meta-analysis. *Pediatric Physical Therapy, 26*(3), 289-300. [doi: 10.1097/PEP.0000000000000046](https://doi.org./10.1097/PEP.0000000000000046)

Chen, H. C., Chen, C. L., Kang, L. J., Wu, C. Y., Chen, F. C., & Hong, W. H. (2014b). Improvement of upper extremity motor control and function after home-based constraint induced therapy in children with unilateral cerebral palsy: Immediate and long-term effects*. Archives of Physical Medicine and Rehabilitation, 95*(8), 1423-1432. [doi: 10.1016/j.apmr.2014.03.025](https://doi.org/10.1016/j.apmr.2014.03.025)

Chen, Y. P., Pope, S., Tyler, D., & Warren, G. L. (2014c). Effectiveness of constraint-induced movement therapy on upper-extremity function in children with cerebral palsy: A systematic review and meta-analysis of randomized controlled trials. *Clinical Rehabilitation, 28*(10), 939-953. [doi](https://doi): 10.1177%2F0269215514544982

Chiu, H. C., Ada, L., & Lee, H. M. (2014). Upper limb training using Wii Sports Resort™ for children with hemiplegic cerebral palsy: A randomized, single-blind trial.*Clinical Rehabilitation, 28*(10), 1015-1024. doi: 10.1177%2F0269215514533709

Christmas, P. M., Sackley, C., Feltham, M. G., & Cummins, C. (2018). A randomized controlled trial to compare two methods of constraint-induced movement therapy to improve functional ability in the affected upper limb in pre-school children with hemiplegic cerebral palsy: CATCH TRIAL. *Clinical Rehabilitation*, *32*(7), 909-918. [doi](https://doi): 10.1177%2F0269215518763512

Cole, K. N., Harris, S. R., Eland, S. F., & Mills, P. E. (1989). Comparison of two service delivery models: In-class and out-of-class therapy approaches. *Pediatric Physical Therapy, 1*(2), 49-54.

Copeland, L., Edwards, P., Thorley, M., Donaghey, S., Gascoigne-Pees, L., Kentish, M., ... & Boyd, R. N. (2014). Botulinum toxin A for nonambulatory children with cerebral palsy: A double blind randomized controlled trial. *The Journal of Pediatrics, 165*(1), 140-146. doi: 10.1016/j.jpeds.2014.01.050

Crompton, J., Imms, C., McCoy, A. T., Randall, M., Eldridge, B., Scoullar, B., & Galea, M. P. (2007). Group-based task-related training for children with cerebral palsy: A pilot study. *Physical & Occupational Therapy in Pediatrics, 27*(4), 43-65. doi: 10.1080/J006v27n04_04

Dagenais, L. M., Lahay, E. R., Stueck, K. A., White, E., Williams, L., & Harris, S. R. (2009). Effects of electrical stimulation, exercise training and motor skills training on strength of children with meningomyelocele: A systematic review. *Physical & Occupational Therapy in Pediatrics, 29*(4), 445-463. doi: 10.3109/01942630903246018

De Vries, D., Beck, T., Stacey, B., Winslow, K., & Meines, K. (2015). Music as a therapeutic intervention with autism: A systematic review of the literature. *Therapeutic Recreation Journal, 49*(3), 220.

Duncan, B., Shen, K., Zou, L. P., Han, T. L., Lu, Z. L., Zheng, H., ... & Caspi, O. (2012). Evaluating intense rehabilitative therapies with and without acupuncture for children with cerebral palsy: A randomized controlled trial. *Archives of Physical Medicine and Rehabilitation, 93*(5), 808-815. doi: 10.1016/j.apmr.2011.12.009

Estes, A., Vismara, L., Mercado, C., Fitzpatrick, A., Elder, L., Greenson, J., ... & Dawson, G. (2014). The impact of parent-delivered intervention on parents of very young children with autism. *Journal of Autism and Developmental Disorders, 44*(2), 353-365. doi: 10.1007/s10803-013-1874-z

Fedewa, A., Davis, M. A., & Ahn, S. (2015). Effects of stability balls on children’s on-task behavior, academic achievement, and discipline referrals: A randomized controlled trial. *American Journal of Occupational Therapy, 69,* 6902220020p1-p9. doi: 10.5014/ajot.2015.014829

Fehlings, D., Novak, I., Berweck, S., Hoare, B., Stott, N. S., & Russo, R. N. (2010). Botulinum toxin assessment, intervention and follow‐up for paediatric upper limb hypertonicity: International consensus statement. *European Journal of Neurology, 17,* 38-56. doi: 10.1111/j.1468-1331.2010.03127.x

Frolek Clark, G. J., & Schlabach, T. L. (2013). Systematic review of occupational therapy interventions to improve cognitive development in children ages birth–5 years. *American Journal of Occupational Therapy, 67*(4), 425-430. doi: 10.1186/1472-6963-7-119

Gringras, P., Green, D., Wright, B., Rush, C., Sparrowhawk, M., Pratt, K., ... & Wiggs, L. (2014). Weighted blankets and sleep in autistic children—A randomized controlled trial. *Pediatrics, 134,* 298-306. doi: 10.1542/peds.2013-4285

Grynszpan, O., Weiss, P. L., Perez-Diaz, F., & Gal, E. (2014). Innovative technology-based interventions for autism spectrum disorders: A meta-analysis. *Autism, 18*(4), 346-361. [doi: 10.1177%2F1362361313476767](https://doi.org/10.1177%2F1362361313476767)

Hahn-Markowitz, J., Berger, I., Manor, I., & Maeir, A. (2017). Impact of the Cognitive–Functional (Cog–Fun) intervention on executive functions and participation among children with attention deficit hyperactivity disorder: A randomized controlled trial. *American Journal of Occupational Therapy, 71*(5), 7105220010p1-9. doi: 10.5014/ajot.2017.022053

Hammond, J., Jones, V., Hill, E. L., Green, D., & Male, I. (2014). An investigation of the impact of regular use of the WiiFit to improve motor and psychosocial outcomes in children with movement difficulties: A pilot study. *Child: Care, Health and Development, 40*(2), 165-175. doi: 10.1111/cch.12029

Hechler, T., Ruhe, A. K., Schmidt, P., Hirsch, J., Wager, J., Dobe, M., ... & Zernikow, B. (2014). Inpatient-based intensive interdisciplinary pain treatment for highly impaired children with severe chronic pain: Randomized controlled trial of efficacy and economic effects. *Pain, 155,* 118-128. doi: 10.1016/j.pain.2013.09.015

Heinrichs, N., Kliem, S., & Hahlweg, K. (2014). Four-year follow-up of a randomized controlled trial of Triple P group for parent and child outcomes. *Prevention Science, 15*(2), 233-245. doi: 10.1007/s11121-012-0358-2

Hoare, B. J., & Imms, C. (2004). Upper-limb injections of botulinum toxin-A in children with cerebral palsy: A critical review of the literature and clinical implications for occupational therapists. *American Journal of Occupational Therapy, 58*(4), 389-397. doi: 10.5014/ajot.58.4.389

Hoare, B., Imms, C., Carey, L., & Wasiak, J. (2007). Constraint-induced movement therapy in the treatment of the upper limb in children with hemiplegic cerebral palsy: A Cochrane systematic review*. Clinical Rehabilitation, 21*(8), 675-685. doi: 10.1002/14651858.cd004149.pub2

Hoare, B. J., Wallen, M. A., Imms, C., Villanueva, E., Rawicki, H. B., & Carey, L. (2010). Botulinum toxin A as an adjunct to treatment in the management of the upper limb in children with spastic cerebral palsy (UPDATE). *Cochrane Database of Systematic Reviews, 1,* CD003469. doi: 10.1002/14651858.cd003469.pub4

Hoy, M. M., Egan, M. Y., & Feder, K. P. (2011). A systematic review of interventions to improve handwriting. *Canadian Journal of Occupational Therapy, 78*(1), 13-25. doi: 10.2182/cjot.2011.78.1.3

Huang, H. H., Fetters, L., Hale, J., & McBride, A. (2009). Bound for success: A systematic review of constraint-induced movement therapy in children with cerebral palsy supports improved arm and hand use. *Physical Therapy, 89*(11), 1126-1141. doi: 10.2522/ptj.20080111

Huang, J. S., Dillon, L., Terrones, L., Schubert, L., Roberts, W., Finklestein, J., ... & Patrick, K. (2014). Fit4Life: A weight loss intervention for children who have survived childhood leukemia. *Pediatric Blood & Cancer, 61*(5), 894-900. [doi: 10.1002/pbc.24937](https://doi.org/10.1002/pbc.24937)

Inguaggiato, E., Sgandurra, G., Perazza, S., Guzzetta, A., & Cioni, G. (2013). Brain reorganization following intervention in children with congenital hemiplegia: A systematic review. *Neural Plasticity*. doi: 10.1155/2013/356275

Jackman, M., Novak, I., & Lannin, N. (2014). Effectiveness of hand splints in children with cerebral palsy: A systematic review with meta‐analysis. *Developmental Medicine & Child Neurology, 56*(2), 138-147. doi: 10.1111/dmcn.12205

Jackman, M., Novak, I., Lannin, N., Froude, E., Miller, L., & Galea, C. (2018). Effectiveness of Cognitive Orientation to daily Occupational Performance over and above functional hand splints for children with cerebral palsy or brain injury: A randomized controlled trial. *BMC Pediatrics, 18*(1), 248. doi: 10.1186/s12887-018-1213-9

James, S., Ziviani, J., Ware, R. S., & Boyd, R. N. (2015). Randomized controlled trial of web‐based multimodal therapy for unilateral cerebral palsy to improve occupational performance. *Developmental Medicine & Child Neurology, 57*(6), 530-538. doi: 10.1111/dmcn.12705

Janeslätt, G., Kottorp, A., & Granlund, M. (2014). Evaluating intervention using time aids in children with disabilities. *Scandinavian Journal of Occupational Therapy, 21*(3), 181-190. doi: 10.3109/11038128.2013.870225

Jones, D. J., Forehand, R., Cuellar, J., Parent, J., Honeycutt, A., Khavjou, O., ... & Newey, G. A. (2014). Technology-enhanced program for child disruptive behavior disorders: Development and pilot randomized control trial. *Journal of Clinical Child & Adolescent Psychology, 43*(1), 88-101. doi: 10.1080/15374416.2013.822308

Kamps, D., Thiemann-Bourque, K., Heitzman-Powell, L., Schwartz, I., Rosenberg, N., Mason, R., & Cox, S. (2015). A comprehensive peer network intervention to improve social communication of children with autism spectrum disorders: A randomized trial in kindergarten and first grade. *Journal of Autism and Developmental Disorders, 45*(6), 1809-1824. doi: 10.1007/s10803-014-2340-2

Kasari, C., Dean, M., Kretzmann, M., Shih, W., Orlich, F., Whitney, R., ... & King, B. (2016). Children with autism spectrum disorder and social skills groups at school: A randomized trial comparing intervention approach and peer composition. *Journal of Child Psychology and Psychiatry, 57*(2), 171-179. doi: 10.1111/jcpp.12460

Katalinic, O. M., Harvey, L. A., Herbert, R. D., Moseley, A. M., Lannin, N. A., & Schurr, K. (2010). Stretch for the treatment and prevention of contractures. *Cochrane Database of Systematic Reviews, 9*(9). doi: 10.1002/14651858.cd007455.pub2

Kaya Kara, O., Atasavun Uysal, S., Turker, D., Karayazgan, S., Gunel, M. K., & Baltaci, G. (2015). The effects of Kinesio Taping on body functions and activity in unilateral spastic cerebral palsy: a single‐blind randomized controlled trial. *Developmental Medicine & Child Neurology, 57*(1), 81-88. doi: 10.1111/dmcn.12583

Krisanaprakornkit, T., Ngamjarus, C., Witoonchart, C., & Piyavhatkul, N. (2010). Meditation therapies for attention‐deficit/hyperactivity disorder (ADHD).  *Cochrane Database of Systematic Reviews, 6*, CD006507. doi: 10.1002/14651858.cd006507.pub2

Kurowski, B. G., Wade, S. L., Kirkwood, M. W., Brown, T. M., Stancin, T., & Taylor, H. G. (2014). Long-term benefits of an early online problem-solving intervention for executive dysfunction after traumatic brain injury in children: A randomized clinical trial. *JAMA Pediatrics, 168*(6), 523-531. doi: 10.1001/jamapediatrics.2013.5070

Lannin, N., Scheinberg, A., & Clark, K. (2006). AACPDM systematic review of the effectiveness of therapy for children with cerebral palsy after botulinum toxin A injections. *Developmental Medicine and Child Neurology, 48*(6), 533-539. doi: 10.1111/j.1469-8749.2006.tb01309.x

Lannin, N. A., Novak, I., & Cusick, A. (2007). A systematic review of upper extremity casting for children and adults with central nervous system motor disorders. *Clinical Rehabilitation, 21*(11), 963-976. doi: 10.1177/0269215507079141

Lidman, G., Nachemson, A., Peny‐Dahlstrand, M., & Himmelmann, K. (2015). Botulinum toxin A injections and occupational therapy in children with unilateral spastic cerebral palsy: a randomized controlled trial. *Developmental Medicine & Child Neurology, 57*(8), 754-761. doi: 10.1111/dmcn.12739

Lin, H. C., & Wuang, Y. P. (2012). Strength and agility training in adolescents with Down syndrome: A randomized controlled trial. *Research in Developmental Disabilities, 33*(6), 2236-2244. doi: 10.1016/j.ridd.2012.06.017

Madlinger-Lewis, L., Reynolds, L., Zarem, C., Crapnell, T., Inder, T., & Pineda, R. (2014). The effects of alternative positioning on preterm infants in the neonatal intensive care unit: A randomized clinical trial. *Research in Developmental Disabilities, 35*(2), 490-497. doi: 10.1016/j.ridd.2013.11.019

Maeir, A., Fisher, O., Bar-Ilan, R. T., Boas, N., Berger, I., & Landau, Y. E. (2014). Effectiveness of Cognitive–Functional (Cog–Fun) occupational therapy intervention for young children with attention deficit hyperactivity disorder: A controlled study. *American Journal of Occupational Therapy, 68*(3), 260-267. doi: 10.5014/ajot.2014.011700

Malow, B. A., Adkins, K. W., Reynolds, A., Weiss, S. K., Loh, A., Fawkes, D., ... & Clemons, T. (2014). Parent-based sleep education for children with autism spectrum disorders. *Journal of Autism and Developmental Disorders, 44*(1), 216-228. doi: 10.1007/s10803-013-1866-z

Mandich, A. D., & Rodger, S. (2006). Dong, being and becoming: Their importance for children. In S. Rodger & J. Ziviani (Eds.), *Occupational therapy with children: Understanding children’s occupations and enabling participation* (pp. 115-117). Oxford: Blackwell Publishing Ltd.

Maskell, J., Newcombe, P., Martin, G., & Kimble, R. (2014). Psychological and psychosocial functioning of children with burn scarring using cosmetic camouflage: A multi-centre prospective randomised controlled trial. *Burns, 40*(1), 135-149. doi: 10.1016/j.burns.2013.04.025

Mast, J. E., Antonini, T. N., Raj, S. P., Oberjohn, K. S., Cassedy, A., Makoroff, K. L., & Wade, S. L. (2014). Web-based parenting skills to reduce behavior problems following abusive head trauma: A pilot study. *Child Abuse & Neglect, 38*(9), 1487-1495. doi: 10.1016/j.chiabu.2014.04.012

Matute-Llorente, Á., González-Agüero, A., Gómez-Cabello, A., Vicente-Rodríguez, G., & Mallén, J. A. C. (2014). Effect of whole-body vibration therapy on health-related physical fitness in children and adolescents with disabilities: A systematic review. *Journal of Adolescent Health, 54*(4), 385-396. doi: 10.1016/j.jadohealth.2013.11.001

McLean, B., Taylor, S., Blair, E., Valentine, J., Carey, L., & Elliott, C. (2017). Somatosensory discrimination intervention improves body position sense and motor performance in children with hemiplegic cerebral palsy. *American Journal of Occupational Therapy, 71*(3), 7103190060p1-9. doi: 10.5014/ajot.2016.024968

Meany‐Walen, K. K., Bratton, S. C., & Kottman, T. (2014). Effects of Adlerian play therapy on reducing students' disruptive behaviors. *Journal of Counseling & Development, 92*(1), 47-56. doi: 10.1002/j.1556-6676.2014.00129.x

Miller-Kuhaneck, H., & Watling, R. (2018). Parental or teacher education and coaching to support function and participation of children and youth with sensory processing and sensory integration challenges: A systematic review. *American Journal of Occupational Therapy, 72*(1), 7201190030p1-11. doi: 10.5014/ajot.2018.029017

Montero, S. M., & Gómez-Conesa, A. (2014). Technical devices in children with motor disabilities: A review. *Disability and Rehabilitation: Assistive Technology, 9*(1), 3-11. doi: 10.3109/17483107.2013.785034

Morgan, C., Novak, I., Dale, R. C., & Badawi, N. (2015). Optimising motor learning in infants at high risk of cerebral palsy: A pilot study. *BMC Pediatrics, 15*(1), 30. doi: 10.1186/s12887-015-0347-2

Morgan, C., Darrah, J., Gordon, A. M., Harbourne, R., Spittle, A., Johnson, R., & Fetters, L. (2016). Effectiveness of motor interventions in infants with cerebral palsy: A systematic review. *Developmental Medicine & Child Neurology, 58*(9), 900-909. doi: 10.1111/j.1365-2214.2008.00921_2.x

Morgan, C., Novak, I., Dale, R. C., Guzzetta, A., & Badawi, N. (2016b). Single blind randomised controlled trial of GAME (Goals-Activity-Motor Enrichment) in infants at high risk of cerebral palsy. *Research in Developmental Disabilities, 55,* 256-267. doi: 10.1186/s12883-014-0203-2

Novak, I. (2014a). Evidence to practice commentary new evidence in coaching interventions. *Physical & Occupational Therapy in Pediatrics, 34*(2), 132-137. doi: 10.3109/01942638.2014.903060

Novak, I., & Berry, J. (2014b). Home program intervention effectiveness evidence. *Physical & Occupational Therapy in Pediatrics, 34*(4), 384-389. doi: 10.3109/01942638.2014.964020

Park, H. Y., Maitra, K., Achon, J., Loyola, E., & Rincón, M. (2014). Effects of early intervention on mental or neuromusculoskeletal and movement-related functions in children born low birthweight or preterm: A meta-analysis. *American Journal of Occupational Therapy, 68*(3), 268-276. doi: 10.5014/ajot.2014.010371

Pfeiffer B, Clark GF, & Arbesman, M. (2018). Effectiveness of cognitive and occupation-based interventions for children with challenges in sensory processing and integration: A systematic review. *American Journal of Occupational Therapy, 72*(1), 7201190020p1-7201190020p9. doi: 10.5014/ajot.2018.028233

Polatajko, H. J., & Cantin, N. (2010). Exploring the effectiveness of occupational therapy interventions, other than the sensory integration approach, with children and adolescents experiencing difficulty processing and integrating sensory information. *American Journal of Occupational Therapy, 64*(3), 415-429. doi: 10.5014/ajot.2010.09072

Reeuwijk, A., van Schie, P. E., Becher, J. G., & Kwakkel, G. (2006). Effects of botulinum toxin type A on upper limb function in children with cerebral palsy: A systematic review. *Clinical Rehabilitation, 20*(5), 375-387. doi: 10.1191/0269215506cr956oa

Sakzewski, L., Lewis, M. J., McKinlay, L., Ziviani, J., & Boyd, R. N. (2016). Impact of multi‐modal web‐based rehabilitation on occupational performance and upper limb outcomes: Pilot randomized trial in children with acquired brain injuries.*Developmental Medicine & Child Neurology, 58*(12), 1257-1264. doi: 10.1111/dmcn.13157

Schaaf, R. C., Dumont, R. L., Arbesman, M., & May-Benson, T. A. (2018). Efficacy of occupational therapy using Ayres Sensory Integration®: A systematic review. *American Journal of Occupational Therapy, 72*(1), 7201190010p1-10. doi: 10.5014/ajot.2018.028431

Smith, T. O., Bacon, H., Jerman, E., Easton, V., Armon, K., Poland, F., & Macgregor, A. J. (2014). Physiotherapy and occupational therapy interventions for people with benign joint hypermobility syndrome: A systematic review of clinical trials. *Disability and Rehabilitation, 36*(10), 797-803. doi: 10.3109/09638288.2013.819388

Snider, L., Majnemer, A., & Darsaklis, V. (2010). Virtual reality as a therapeutic modality for children with cerebral palsy. *Developmental Neurorehabilitation, 13*(2), 120-128. doi: 10.3109/17518420903357753

Speth, L., Janssen-Potten, Y., Rameckers, E., Defesche, A., Winkens, B., Becher, J., ... & Vles, H. (2015). Effects of botulinum toxin A and/or bimanual task-oriented therapy on upper extremity activities in unilateral cerebral palsy: A clinical trial. *BMC Neurology, 15*(1), 143. doi: 10.1186/s12883-015-0404-3

Spittle, A., Orton, J., Doyle, L. W., & Boyd, R. (2007). Early developmental intervention programs post hospital discharge to prevent motor and cognitive impairments in preterm infants. *Cochrane Database of Systematic Reviews*, (2), CD005495. doi: 10.1002/14651858.cd005495.pub2

Spittle, A., Orton, J., Anderson, P., Boyd, R., & Doyle, L. W. (2012). Early developmental intervention programmes post-hospital discharge to prevent motor and cognitive impairments in preterm infants. *Cochrane Database of Systematic Reviews,* (12), CD005495. doi: 10.1002/14651858.cd005495.pub3

Stavness, C. (2006). The effect of positioning for children with cerebral palsy on upper-extremity function: A review of the evidence. *Physical & Occupational Therapy in Pediatrics, 26*(3), 39-53. doi: 10.1300/j006v26n03_04

Stickles Goods, K., Ishijima, E., Chang, Y. C., & Kasari, C. (2013). Preschool based JASPER intervention in minimally verbal children with autism: Pilot RCT. *Journal of Autism and Developmental Disorders, 43*(5), 1050-1056. doi: 10.1007/s10803-012-1644-3

Storebø, O. J., Skoog, M., Damm, D., Thomsen, P. H., Simonsen, E., & Gluud, C. (2011). Social skills training for Attention Deficit Hyperactivity Disorder (ADHD) in children aged 5 to 18 years. *Cochrane Database of Systematic Reviews, 12,* CD008223. doi: 10.1002/14651858.cd008223.pub2

Tatla, S. K., Sauve, K., Virji‐Babul, N., Holsti, L., Butler, C., & Van Der Loos, H. F. M. (2013). Evidence for outcomes of motivational rehabilitation interventions for children and adolescents with cerebral palsy: An American Academy for Cerebral Palsy and Developmental Medicine systematic review. *Developmental Medicine & Child Neurology, 55*(7), 593-601. doi: 10.1111/dmcn.12147

Tatla, S. K., Sauve, K., Jarus, T., Virji-Babul, N., & Holsti, L. (2014). The effects of motivating interventions on rehabilitation outcomes in children and youth with acquired brain injuries: A systematic review. *Brain Injury, 28*(8), 1022-1035. doi: 10.3109/02699052.2014.890747

Vargas, S., & Lucker, J. R. (2016). A quantitative summary of The Listening Program (TLP) efficacy studies: What areas were found to improve by TLP intervention? *Occupational Therapy International, 23*(2), 206-217. doi: 10.1002/oti.1425

Vroland‐Nordstrand, K., Eliasson, A. C., Jacobsson, H., Johansson, U., & Krumlinde‐Sundholm, L. (2016). Can children identify and achieve goals for intervention? A randomized trial comparing two goal‐setting approaches. *Developmental Medicine & Child Neurology, 58*(6), 589-596. doi: 10.1111/dmcn.12925

Wallen, M. M., & Gillies, D. (2006). Intra‐articular steroids and splints/rest for children with juvenile idiopathic arthritis and adults with rheumatoid arthritis. *Cochrane Database of Systematic Reviews, 1,* CD002824. doi: 10.1002/14651858.cd002824.pub2

Wells, H., Marquez, J., & Wakely, L. (2018). Garment therapy does not improve function in children with cerebral palsy: A systematic review. *Physical & Occupational Therapy in Pediatrics*, 38, 395-416. doi: 10.1080/01942638.2017.1365323

Westendorp, M., Houwen, S., Hartman, E., Mombarg, R., Smith, J., & Visscher, C. (2014). Effect of a ball skill intervention on children’s ball skills and cognitive functions. *Med. Sci. Sports Exerc, 46*, 414-422. doi: 10.1249/mss.0b013e3182a532b3

Whalen, C. N., & Case-Smith, J. (2012). Therapeutic effects of horseback riding therapy on gross motor function in children with cerebral palsy: A systematic review.*Physical & Occupational Therapy in Pediatrics, 32*(3), 229-242. doi: 10.3109/01942638.2011.619251

Willis, C., Nyquist, A., Girdler, S., Jahnsen, R., Reid, S., Rosenberg, M., ... & Elliott, C. (2016). Identifying and operationalising the active ingredients of a participation intervention for children with disabilities: Staff perspectives. *Developmental Medicine & Child Neurology, 58,* 42-43.

Xu, K., He, L., Mai, J., Yan, X., & Chen, Y. (2015). Muscle recruitment and coordination following constraint-induced movement therapy with electrical stimulation on children with hemiplegic cerebral palsy: A randomized controlled trial. *PloS One, 10*(10), e0138608. doi: 10.1371/journal.pone.0138608

Zadnikar, M., & Kastrin, A. (2011). Effects of hippotherapy and therapeutic horseback riding on postural control or balance in children with cerebral palsy: A meta‐analysis. *Developmental Medicine & Child Neurology, 53*(8), 684-691. doi: 10.1111/j.1469-8749.2011.03951.x

Ziviani, J., Feeney, R., Rodger, S., & Watter, P. (2010). Systematic review of early intervention programmes for children from birth to nine years who have a physical disability. *Australian Occupational Therapy Journal, 57*(4), 210-223. doi: 10.1111/j.1440-1630.2010.00850.x

Zwaigenbaum, L., Bauman, M. L., Choueiri, R., Kasari, C., Carter, A., Granpeesheh, D., ... & Pierce, K. (2015). Early intervention for children with autism spectrum disorder under 3 years of age: Recommendations for practice and research. *Pediatrics, 136*(Suppl 1), S60-S81. doi: 10.1542/peds.2014-3667E

Zwicker, J. G., & Mayson, T. A. (2010). Effectiveness of treadmill training in children with motor impairments: An overview of systematic reviews. *Pediatric Physical Therapy, 22*(4), 361-377. doi: 10.1097/pep.0b013e3181f92e54
